# Furfural Valorization to γ‑Valerolactone over Zr/Sn Zeolite-Supported Catalysts in a Liquid-Phase Continuous Flow Reactor

**DOI:** 10.1021/acs.energyfuels.5c05628

**Published:** 2025-12-23

**Authors:** Vittoria Saraceni, Anna Saotta, Adrián García, Alessandro Allegri, Giuseppe Fornasari, Benjamin Solsona, Nikolaos Dimitratos, Stefania Albonetti

**Affiliations:** 1 Department of Industrial Chemistry “Toso Montanari”, Center for Chemical Catalysis − C3, Alma Mater Studiorum − Università di Bologna, Via Piero Gobetti 85, Bologna 40129, Italy; 2 Department of Chemical Engineering (ETSE), 16781 Universitat de València, Av. Universitat s/n, Burjassot ,Valencia 46100, Spain

## Abstract

This work presents for the first time the combined effect of Sn and Zr in the conversion of furfural (FU) to γ-valerolactone (GVL) by using a single liquid-phase continuous flow reactor. To address the high costs and environmental impact of this cascade reaction, catalytic transfer hydrogenation is a promising approach, utilizing alcohols as hydrogen donors in place of molecular H_2_. This process, which requires both Lewis and Bro̷nsted acidity, when coupled with heterogeneous catalysts, offers a potentially more cost-effective and environmentally friendly alternative. The production of GVL in one pot has been studied using Sn- and Zr-based catalysts supported on dealuminated zeolite Y. The bimetallic catalyst with a Sn:Zr at. ratio of 1:1 achieved the best performance, reaching a yield to GVL of ca. 45% at 180 °C using 2-propanol as a hydrogen source, with a 10 min contact time. Moreover, stability studies, including long-term catalytic tests under reaction conditions, were carried out to evaluate the durability and the deactivation. In addition, an efficient regeneration protocol was developed and optimized, enabling catalyst reuse across multiple cycles with performance in terms of conversion and selectivities comparable to those observed with the fresh materials.

## Graphical abstract

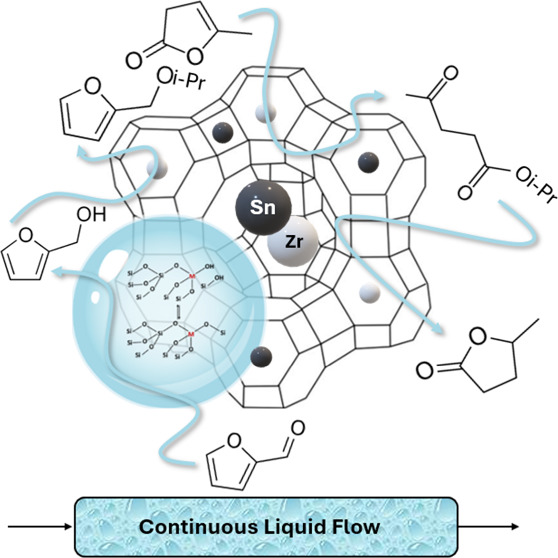

## Introduction

Furfural (FU) is a particularly reactive aldehyde, obtained from the dehydration of the C5 sugars of hemicellulose.^1,2^ Thanks to its reactivity, numerous value-added chemical compounds can be obtained from it.^1–4^ This aldehyde is mainly used to produce furfuryl alcohol (FAL).^5–7^ However, other interesting molecules can be obtained from FU, such as γ-valerolactone (GVL), a five-carbon cyclic ester, currently used mostly as a green solvent due to its high production costs.^8,9^ Nevertheless, it is of great interest due to its potential use as a platform chemical to produce other compounds and biobased fuel. Therefore, research is focusing on developing an economically and environmentally sustainable production process.^1,10–12^ The cascade reaction from FU to GVL involves some reduction steps, which industrially are typically carried out under high pressures of molecular hydrogen and using noble metal-based catalysts, making the process highly impactful on the environment.^13,14^ One alternative to traditional reduction is catalytic transfer hydrogenation (CTH), where molecular hydrogen is substituted with a sacrificial hydrogen donor such as an alcohol, which reduces the substrate.^15–19^ Additionally, alcohol is usually employed as a solvent to shift the equilibrium toward the products. Then, the one-pot reaction can be performed through successive acid-catalyzed steps, some of which are catalyzed by Lewis acidity and others by Bro̷nsted acidity. In this latter characteristic lies the main complexity of the process: developing a bifunctional catalyst, which offers both kinds of acidity.

Among the non-noble metal-based materials, transition metal oxide-based systems supported on zeolites can meet the acidity requirements of the reaction because they exhibit Bro̷nsted acidity arising from the zeolitic support and Lewis acidity characteristic of metal oxides.^20,21^ Previous studies show how zirconium oxide can provide the right Lewis acidity to promote H-transfer reactions.^5,15,22–24^ Instead, materials based on beta- or Y-zeolites have been reported as effective Bro̷nsted catalysts.^9,25–28^ Regarding Y-zeolite-based systems, a significant result was achieved by Zhang et al.,^29^ who designed a catalytic system for converting FU into GVL using a mixture of two distinct solid catalysts: Zr-HY, with prior dealumination of the zeolite, and Al-HY. By employing 2-pentanol as the solvent, a GVL selectivity of 85% was achieved after 5 h at 120 °C in a batch reactor. Additionally, Garcia et al.^30^ reported the transformation of FU into GVL in one pot using Sn- and Zr-based catalysts supported on treated zeolite Y in a batch system. They demonstrated that these materials led to a GVL yield of ca. 80% after 1 h at 180 °C, using 2-propanol as a hydrogen source. The obtained results were attributed to the uniform distribution and the synergistic effect of metals on the support surface, which forms mixed dispersed Zr–O–Sn species, promoting the presence of a proper amount of Lewis and Bro̷nsted acid sites.

Sn- and Zr-based oxide materials supported on Y-zeolite have thus been shown to possess adequate acidic properties for conducting the cascade reaction. For this reason, they were tested in a continuous flow liquid-phase reactor. The latter represents an innovation in this field as, in the literature, this cascade reaction is primarily studied in discontinuous batch systems.^31–34^ Demonstrating the feasibility of the process in a continuous phase could lead to well-known advantages, as higher productivity, efficiency, and sustainability compared to batch systems, also enabling optimal control of the reaction conditions.^8,35–38^ Few reports on the production of GVL in continuous flow have been reported, for example, Zhao et al.^39^ obtained a GVL yield of ca. 85% from methyl levulinates (ML) using 2-propanol as a hydrogen source at 150 °C, 40 bar on a 5 wt % Ru/C commercial catalyst. Lopéz-Alguado et al.^40^ attempted to obtain GVL directly from FU but obtained fast catalyst deactivation. The same catalysts were, however, effective in converting levulinic acid (LA) to GVL in the continuous liquid phase. The entire cascade reaction has, instead, been successfully performed by Saotta et al.^8^ in a continuous flow reactor on the Ti/Zr/O (1:1) catalyst with a GVL selectivity of ca. 20% at 180 °C using 2-propanol as a hydrogen source, with a 10 min contact time. Moreover, Garcìa et al.^33^ reported an 8% yield into GVL on 1Pt/10Zr/Sep catalyst at 180 °C using 2-propanol as a hydrogen source, with a 10 min contact time in a continuous flow starting from FU. Therefore, it is of great interest to attempt this reaction in a continuous reactor due to the several advantages over traditional batch systems and to investigate materials that lead to improved yields in GVL. The choice of Sn/Zr-mixed oxide supported on zeolite Y as the catalyst for this reaction is also strategic. Zeolites provide a robust, acid-supporting framework, while the transition metal oxide, specifically zirconium, contributes the required Lewis acidity for efficient hydrogen transfer. Among various zeolites, zeolite Y has been selected due to its large pore size (∼7.4 Å),^41^ which facilitates the diffusion of organic substrates and intermediates. Crucially, it has been recently reported that a controlled dealumination process can be used to tailor the local coordination environment of aluminum sites, resulting in enhanced anchoring sites for species such as Zr^4^^+^ and Sn^4^^+^, promoting the generation of catalytically active sites suitable for CTH reductions.^42^ As a result, the integration of a continuous flow reactor with a Sn/Zr oxide on the zeolite catalyst presents a novel and effective method for this transformation, offering significant potential for scaling up the process for industrial use.

In this study, a systematic approach was adopted to investigate and optimize experimental parameters to elucidate the structure–activity relationship of the catalytic system. This strategy allowed for the optimization of product yields as well as a comprehensive assessment of catalyst stability and deactivation phenomena. Particular attention was devoted to the identification of effective regeneration strategies, which are essential for prolonging the catalyst lifetime and maintaining the overall sustainability and efficiency of the catalytic process.

## Experimental Section

### Materials

Sn:Zr/HY (A:B) were used to study the cascade reaction from furfural to GVL. The substrates used to examine the one-pot reaction were furfural (Sigma-Aldrich, 98%), furfuryl alcohol (Sigma-Aldrich, 98%), furfuryl ethyl ether (Sigma-Aldrich, 98%), α-angelica lactone (Sigma-Aldrich, 98%), propyl levulinate (Sigma-Aldrich, 95%), and γ-valerolactone (Sigma-Aldrich, 99%). Isopropanol (Sigma-Aldrich, 99.5%) was used as a solvent and octane (Sigma-Aldrich, 99%) as a standard for GC-FID analysis. SiC (silicon carbide) pellets were used as diluents for the continuous flow reactor.

### Synthesis and Characterization of Catalysts

The catalysts used are oxides and mixed oxides based on Zr and Sn supported on zeolite Y. Oxide synthesis was performed by direct calcination of precursors (zirconium oxynitrate and tin oxalate) in static air for 6 h at 500 °C. The oxides were then supported on the zeolite dealuminated by acid treatment with HNO_3_ in an aqueous solution as described by Garcia et al.^30^ Briefly, zeolite Y with a Si/Al ratio of 15 was dealuminated using a nitric acid aqueous solution at 80 °C for 24 h. After treatment, the mixture was cooled, centrifuged to recover the zeolite, washed with deionized water, and then dried in a furnace at 100 °C overnight (Figure 1). The different synthesized composite systems have been identified with the acronym “Sn:Zr/Al-HY (A:B)” where the symbols Sn and Zr indicate, respectively, the presence of tin and zirconium, Al-HY indicates zeolite Y or HY, the dealuminated zeolite, and between brackets the molar ratio between tin and zirconium will be indicated. All of the tested catalysts are reported in Table 1.

**Figure 1:**
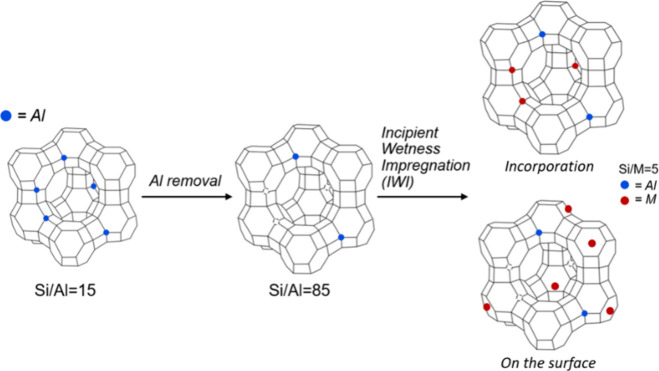
Schematic representation of the dealumination process and replacement of the metal sites.

**Table 1:** Metal Content of the Tested Catalysts

catalyst	molar ratio Sn/Zr	molar ratio Si/Al	molar ratio Si/M
HY		85	
SnO_2_			
Sn/HY		85	5
Sn:Zr/HY (9:1)	9:1	85	5
Sn:Zr/HY (1:1)	1:1	85	5
Sn:Zr/HY (1:9)	1:9	85	5
Zr/HY		85	5
ZrO_2_			

Catalysts’ crystalline phases were identified by X-ray powder diffraction (XRD), using a Bragg/Brentano diffractometer (X’pertPro PANalytical) equipped with an X’Celerator detector and Cu Kα (λ = 1.5418 Å) radiation. The diffractograms were recorded in the 5° < 2θ < 80° range, with a 0.05° 2θ acquisition step and 600 s acquisition time. Textural parameters (*S*_BET_, *V*_P_, and *D*_P_) were assessed via N_2_ adsorption–desorption analysis using a Sorpty 1750 Fison instrument. Before cooling in a liquid nitrogen bath for N_2_ adsorption, samples were outgassed at 200 °C. Surface areas were determined by using the Brunauer–Emmett–Teller (BET) equation assuming a cross-section of 0.162 nm for the nitrogen molecule. The pore size distribution was obtained using the Barrett – Joyner – Halenda (BJH) model.

### Continuous Flow Reactor

Continuous flow reactions were carried out using a homemade liquid-phase fixed-bed reactor (Figure 2). SiC as the desired diluent was loaded into the reactor together with 1 mL of catalyst placed within the isothermal zone of the oven. To facilitate the postreaction separation, before loading the reactor, the diluent was sieved to obtain particles with a diameter higher than 60 mesh, while the catalysts were sieved to have a diameter between 80 and 60 mesh. The reactor was then pressurized with N_2_ at 40 bar, and the reaction mixture was fed into the reactor. These reaction conditions were chosen as they have already been optimized.^8^

**Figure 2:**
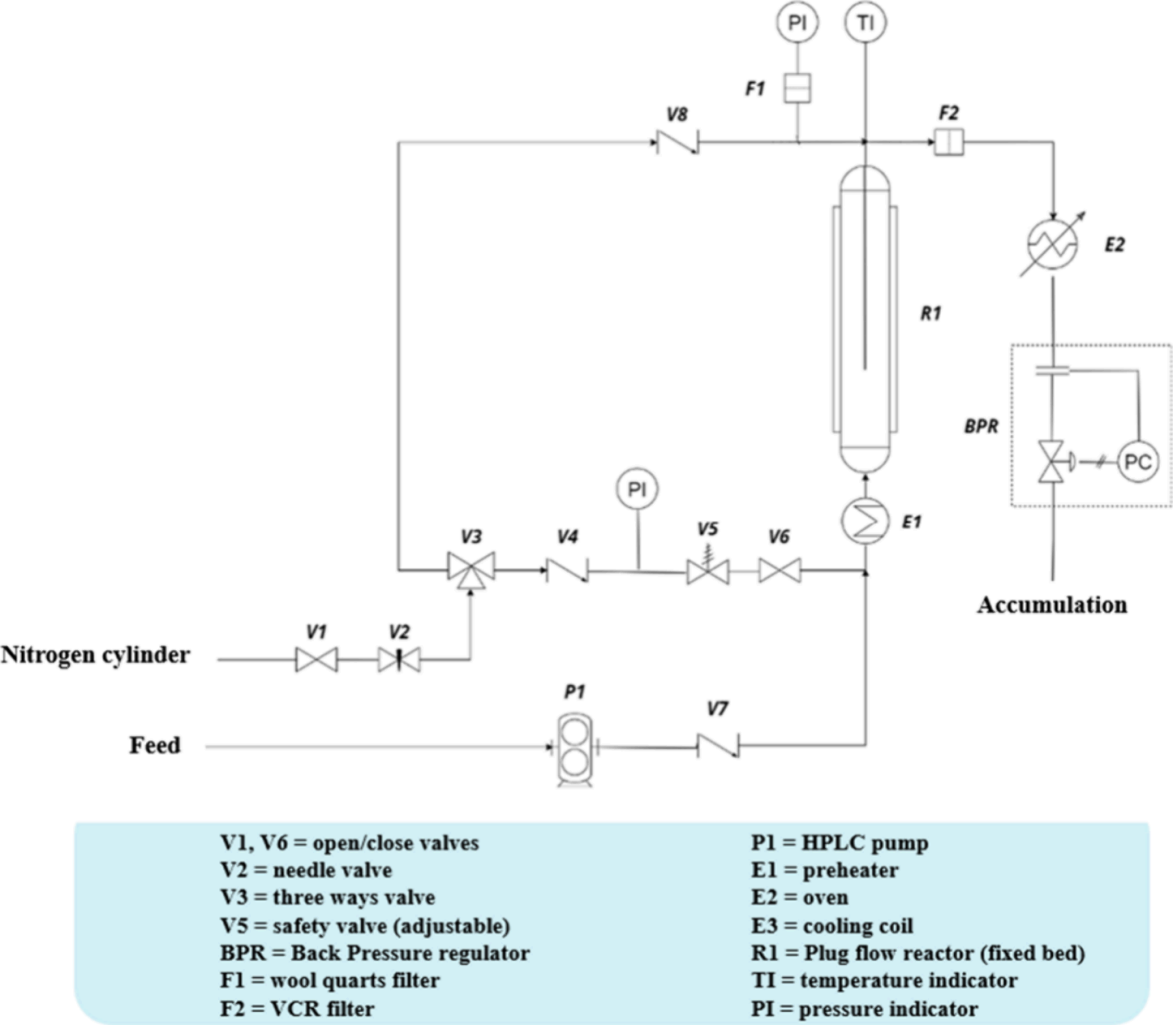
Scheme of the liquid-phase continuous reactor used.

A 67 mM furfural solution in isopropanol, containing 1 equiv of H_2_O and 330 μL of octane, used as an internal standard, was prepared in a 250 mL flask to be used as a feed. The samples were collected every hour in a 10 mL volumetric flask, diluted with isopropanol, and analyzed via gas chromatography (Shimadzu GC 2010 Pro).

The analysis method used was as follows: the injector was heated to 280 °C for the vaporization of the mixture, with a N_2_ flow as an eluent of 1.2 mL/min and split ratio equal to 30; the Agilent HP-5 column (diameter 0.32 mm, length 30 m) was placed inside a heated chamber at a controlled temperature through the following temperature program: 2 min of isotherm at 50 °C, heating of 10 °C/min up to 250 °C, and followed by an isotherm of 2 min at 250 °C; the FID detector was heated to 250 °C for compound detection. To calculate the response factors, the moles, hence the molar flow, and finally, the conversion and selectivity of the different products obtained, calibration curves of the principal commercial molecules involved in the cascade reaction (FU, FAL, α-AnL, β-AnL, FPE, GVL, and IPL) were constructed. Response factors and retention times were identified: 5.2, 5.5, 5.8, 7.0, 7.2, 7.3, and 9.9 min. According to eqs 1–3, furfural conversion, selectivity of products, and the percentage of undesired products (Others) were calculated, respectively:

$$\mathrm{conversion}(\%)=\frac{\left[n{˙}_{\mathrm{FUi}}\left(\frac{\mathrm{mol}}{\min}\right)-{ṅ}_{\mathrm{FUf}}\left(\frac{\mathrm{mol}}{\min}\right)\right]}{{ṅ}_{\mathrm{FUi}}\left(\frac{\mathrm{mol}}{\min}\right)}\times 100$$ 1

$${\mathrm{selectivity}}_{X}(\%)=\frac{{ṅ}_{X}\left(\frac{\mathrm{mol}}{\min}\right)}{\left[{ṅ}_{\mathrm{FUi}}\left(\frac{\mathrm{mol}}{\min}\right)-{ṅ}_{\mathrm{FUf}}\left(\frac{\mathrm{mol}}{\min}\right)\right]}\times 100$$ 2

others(%)=100−∑selectivities 3

*ṅ*_FUi_ *and ṅ*_FUf_ are the initial and final molar flows of furfural (mol/min), while *ṅ_X_* is the molar flow (mol/min) of product *X*. All results are expressed as percentages.

## Results and Discussion

### Catalytic Results

Sn:Zr/HY (A:B) catalysts have been previously tested in a batch reactor, and the corresponding results have been published.^30^ Based on the promising performance observed, we extended our investigation using a continuous liquid-phase fixed-bed reactor to evaluate their catalytic performance in the cascade reaction from FU to GVL (Figure 3) and study specific experimental parameters, the stability of the catalyst, deactivation, and especially regeneration protocols. The reaction network is the following, and it has been thoroughly discussed previously.^8^ The one-pot reaction comprises numerous steps; first, the reduction of FU to furfuryl alcohol (FAL) through the CTH with isopropanol (*i*PrOH). Therefore, Bro̷nsted acidity is required to allow the formation of furfuryl isopropyl ether (FPE), which is likely to be the predominant species given the high concentration of *i*PrOH. The next step involves the hydrolytic ring opening of FPE to isopropyl levulinate (IPL), which can be reduced through another H-transfer reaction to form isopropyl 4-hydroxypentanoate. The high instability of the latter leads to its immediate conversion to GVL. In addition, another parallel reaction is possible. The reaction produces α- and β-angelic lactone (AnL) from FAL. Their double bond reduction produces GVL as well.^8,43^

**Figure 3:**
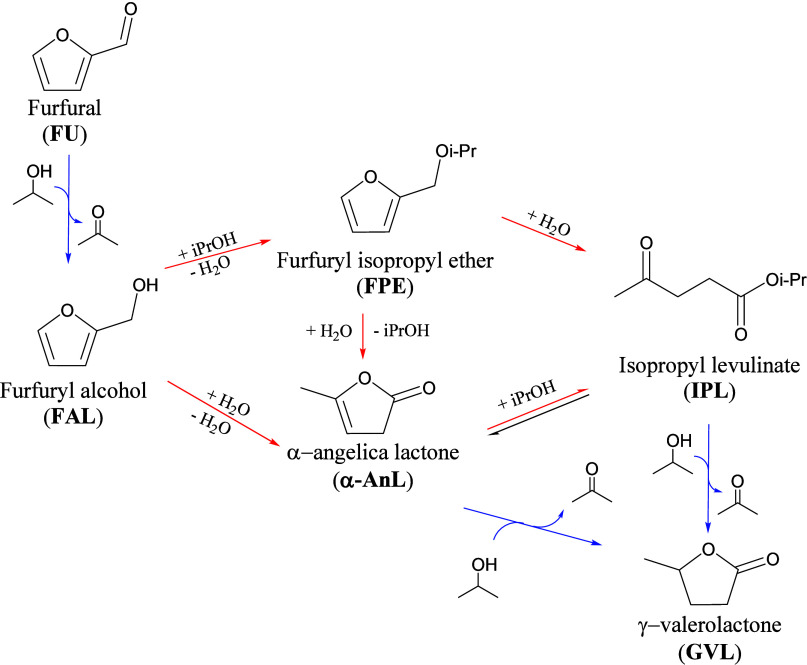
Schematic representation of the cascade mechanism from FU to GVL. The blue arrows indicate the steps catalyzed by Lewis acidity, while the red ones indicate the steps catalyzed by Bro̷nsted acidity.

In addition to the desired products, other compounds such as *trans*-furfurylidenacetone, bis­(furan-2-yl)­methane, and 2,2′-(oxybis­(methylene))­difuran have been identified via GC-MS (gas chromatography–mass spectrometer) after catalytic testing (Figure 4). The first compound arises from the aldol condensation reaction between FU and acetone, which is a coproduct of the CTH reaction. The latter two compounds result from preliminary oligomerization reactions preceding the formation of humins. These products, as well as the heavy carbonaceous species that are formed on the surface of the catalyst, result in the “Others” selectivity reported in the catalytic tests.

**Figure 4:**
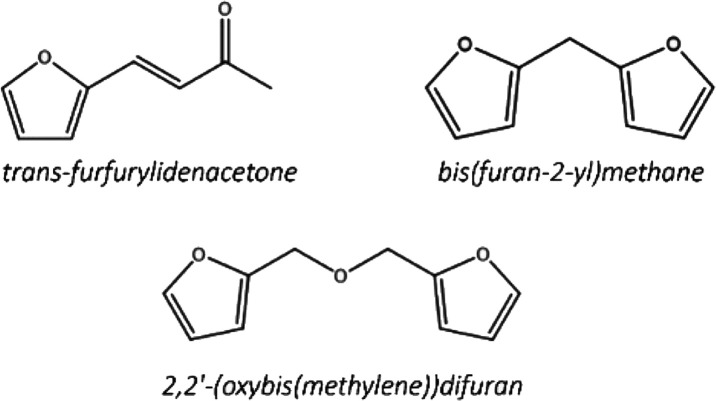
Some of the undesirable products of the cascade reaction from FU to GVL.

First, the zeolitic support HY and the two pure oxides were tested to evaluate the catalytic performance of the individual components of the catalysts presented later. The results are shown in Figure 5a–c, while in Figure 5d, the selectivities and conversion values were an average of the analyses from the steady state to the end of the studied reaction. During the catalytic tests for all catalysts, the trends of FU conversion and selectivity of products have been evaluated as a function of time on stream (i.e., the duration for which the catalytic bed is exposed to the reaction mixture) as shown in Figure 5 for HY, SnO_2_ (Figure 5b), and ZrO_2_ (Figure 5c). The initial point consistently exhibited very low substrate conversion values. This is attributed to the experimental procedure: the initial accumulation occurs when the furnace reaches a temperature of 180 °C, at which point the system has not yet reached the steady state. Once the steady state was reached, the trends were constant for over 6 h as it is shown by the selectivity values that varied at most 4% during the reaction.

**Figure 5:**
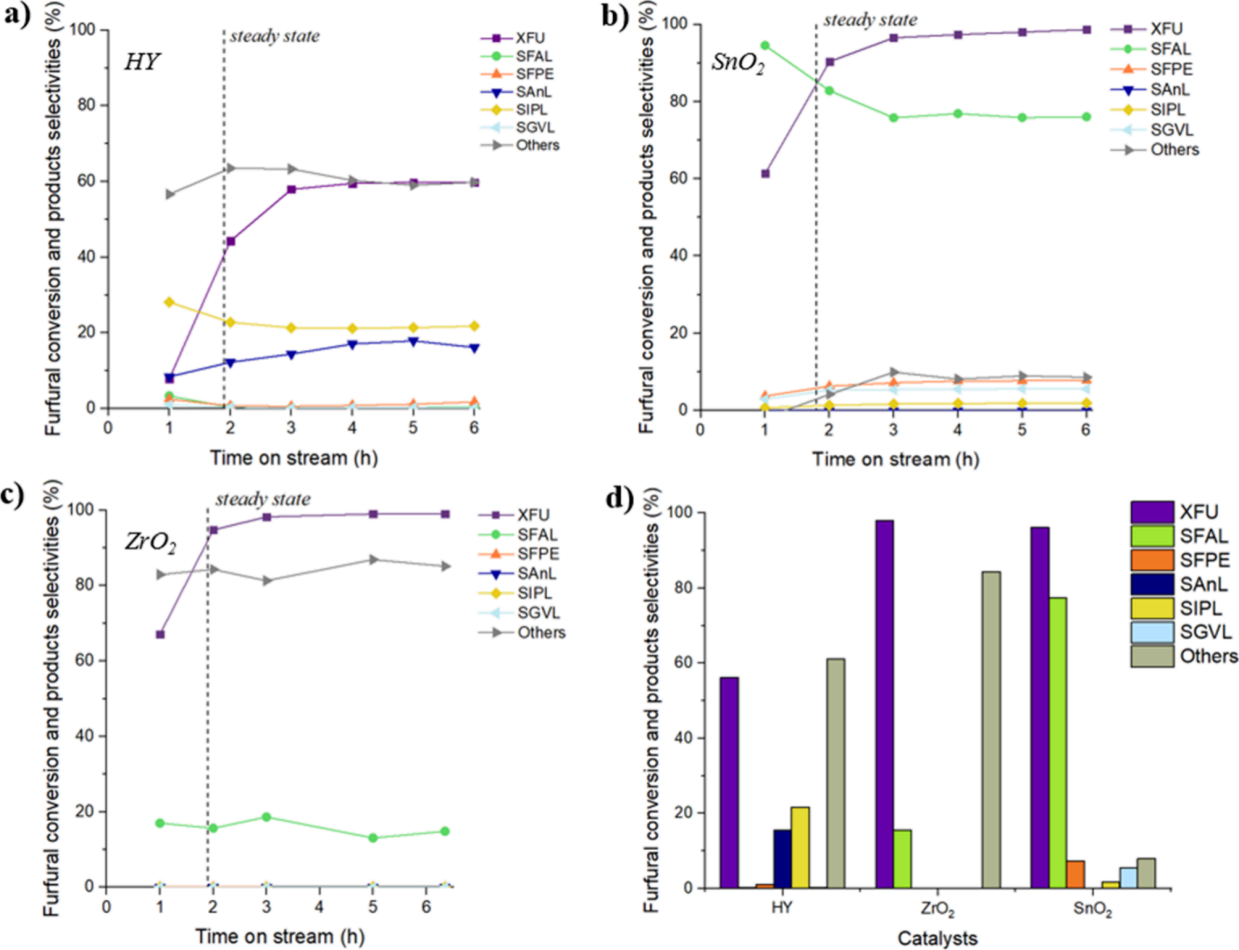
FU conversion and selectivity of products as a function of time (h), reaction conditions: [FU] = 67 mM, 1 equiv of H_2_O, τ=10 min, *T* = 180 °C on (a) HY mcat = 0.4600 g, (b) SnO_2_ mcat = 1.6127 g, and (c) ZrO_2_ mcat = 0.9063 g. (d) Comparison of catalytic average results of the support and the two pure oxides in the cascade reaction from FU to GVL.

Using zeolite Y in the reaction, FU conversion of 56% was detected, but no yield of GVL was observed. The predominant product is IPL, with a selectivity of 22%. Based on the acidity data, the BAS/LAS ratio was found to be 0.02, indicating that the dealuminated zeolite contains a very low concentration of Bro̷nsted acid sites, which are crucial for the activation and opening of the furanic ring. Moreover, the number of Lewis acid sites is also insufficient to effectively promote the desired reaction pathway. This can be attributed to the low amount of Lewis acid sites or the lack of sufficiently strong Lewis acid sites; in fact, pyridine adsorption analysis reveals the presence of predominantly weak acid sites.^30^ This limits the conversion of IPL or AnL into GVL, since, similar to the first step from FU to FAL, the last step also involves a CTH reaction but likely requires a greater acidity strength. The selectivity toward lower-value products (“Others”) reached 61%, as the low conversion rate of FU allowed more time for oligomerization reactions to occur.

The two pure oxides (SnO_2_ and ZrO_2_) were also tested in the same conditions (Figure 5b,c). Regarding ZrO_2_, the FU conversion is 98% and the predominant product is FAL. However, the selectivity toward FAL was low, approximately 16%, which is likely due to the crystalline phase of zirconia, which, following a synthesis via direct calcination of the precursors, is primarily monoclinic^30^ as reported in the XRD analysis presented in Figure S11. The presence of this phase results in a very low surface area of 57 m^2^g^–1^ (Table 2), affecting the catalytic activity of the material and its selectivity toward other products. Indeed, the selectivity toward FAL was much lower compared to the C-loss value that reaches 84%, suggesting that this type of system likely favors secondary reactions, like alcohol condensations and oligomerizations, that did not allow high selectivity toward FAL. On the contrary, a tetragonal zirconia would have reached higher selectivity in FAL and low C-loss, as previously demonstrated.^8^ In addition, the catalyst lacks Bro̷nsted acidity to catalyze the subsequent steps after the alcohol formation.^16,44^

**Table 2:** Physiochemical Characteristics of the Zr- and Sn-Based Catalysts Obtained via N_2_-Adsorption/Desorption (Figures S1, S2, S6, S7, S9, S10, S12, S13, S15, and S17 and Table S1) and XRD (Figures S5, S8, S11, S14, S16, S18, and S19)­^a^

	surface area (m^2^/g)		crystallite size SnO_2_ (nm)		crystallite size ZrO_2_ (nm)			
catalyst	fresh	spent	fresh	spent	fresh	spent	total acidity (μmol/g)	BAS/LAS
HY	999	780					231.9	0.020
SnO_2_	22	22	14.2	25.9				
Sn/HY	715	492	10.0	13.3			368.6	0.104
Sn:Zr/HY (9:1)	703	n.d.	9.4	16.3	n.d.	n.d.	805.2	0.118
Sn:Zr/HY (1:1)	730	592	n.d.	n.d.	n.d.	n.d.	530.6	0.187
Sn:Zr/HY (1:9)	774	n.d.	n.d.		5.6	31.3	624.9	0.032
Zr/HY	802	630			6.2	34.7	259.0	0.007
ZrO_2_	59	57			9.0	14.5		

a n.d.: not detected. FTIR-py adapted from ref 30.

Concerning SnO_2_, an FU conversion of 98% was reached, and the main product was FAL with a yield of 77%. The results clearly indicate that the oxide exhibits predominantly Lewis acid sites but also shows the presence of Bro̷nsted acidity.^28,45^ The presence of BAS enabled the conversion of FAL into subsequent products like FPE, IPL, and GVL, even if in low percentages, respectively, 7%, 2%, and 5%. Furthermore, a significant difference between the two C-loss values of SnO_2_ and ZrO_2_ was observable, indicating that secondary reactions were less favored in the Sn-based catalyst system, reaching only 9% yield into secondary products.

Subsequent tests were carried out, extending the study to all of the other catalysts reported in Table 2. The results are summarized in Figure 6. Regarding the monometallic catalysts, Sn/HY and Zr/HY, the best performances were achieved with the Sn-based catalyst (Figures S3 and S4) with an FU conversion of 96% and a 35% selectivity in GVL. Sn/HY exhibited a higher density of total acid sites, 368.6 μmol g^–1^, including a higher quantity of strong acid sites.^30^ The BAS/LAS ratio for this catalyst has a value of 0.10, much higher than the value of the Zr/HY catalyst, which has a BAS/LAS ratio of 0.01 (Table 2). Such a low ratio is attributed to the very low quantity of Bro̷nsted acid sites in Zr/HY, only 1.8 μmol g^–1^, which does not allow the complete conversion of all intermediates (FPE and AnL) into IPL. Instead, Sn/HY maintains a better balance between Lewis and Bro̷nsted acidity, resulting in a higher selectivity of GVL and nearly complete conversion of FPE and AnL. However, it is observed in this catalyst as well that there is still a lack of sufficient Lewis acidity (327.9 μmol g^–1^) to convert all IPL and AnL into GVL.

**Figure 6:**
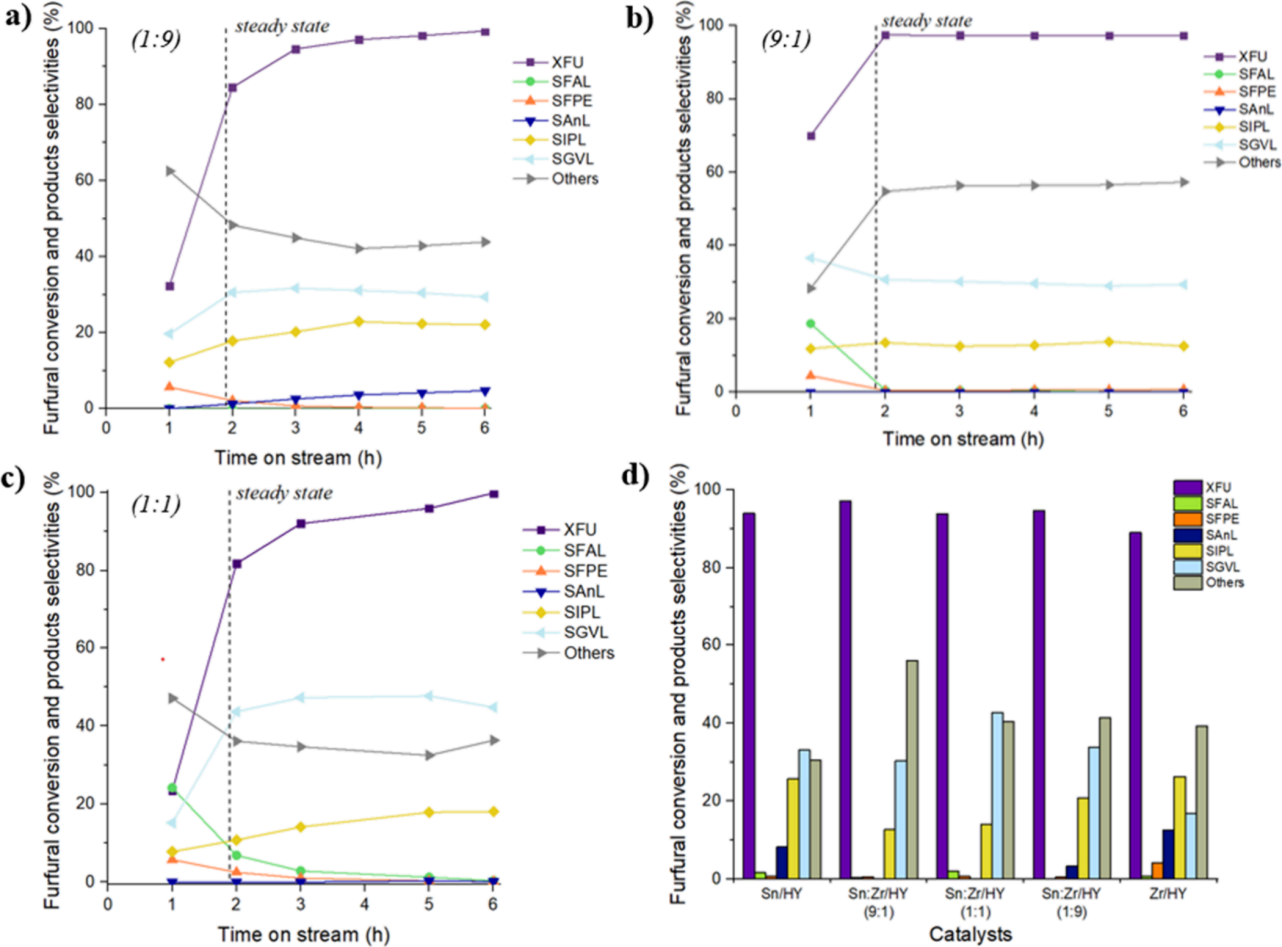
FU conversion and selectivity of products as a function of time (h), reaction conditions: [FU] = 67 mM, 1 equiv of H_2_O, τ = 10 min, *T* = 180 °C on (a) Sn:Zr/HY (1:9) mcat = 0.6000 g, (b) Sn:Zr/HY (9:1) mcat = 0.8921 g, and (c) Sn:Zr/HY (1:1) mcat = 0.5758 g. (d) Comparison of catalytic average results of supported catalysts on HY in the cascade reaction from FU to GVL.

Using bimetallic systems (Sn:Zr/HY (9:1), (1:1), and (1:9)), a synergistic effect emerged, highlighting better results than those obtained on the monometallic systems previously tested for cascade reaction in a batch system.^30^ Similar results were obtained for all three bimetallic catalysts, with an FU conversion of >95%. It is noted that Sn:Zr/HY (9:1), despite having the highest number of total acid sites, 805.2 μmol g^–1^ (Table 2), exhibited lower selectivity toward GVL and IPL, 30% and 13%, respectively, compared to Sn:Zr/HY (1:1) with a yield into GVL of ca. 45% and Sn:Zr/HY (1:9) with 35% of GVL selectivity. This suggests that, although the reaction requires a certain acidity, the BAS/LAS ratio is the fundamental parameter to have a successful process, as demonstrated by previous studies^30^ (Table 2). Additionally, it should be considered that Sn:Zr/HY (9:1) is characterized by the presence of a significant number of weak acid sites,^30^ which are not suitable for the cascade reaction but rather promote secondary reactions, leading to such a high “Others” selectivity of 56%. The best performance was achieved with the Sn:Zr/HY (1:1) catalyst, which results in a GVL selectivity of ca. 45%. The fact that this catalyst has a higher density of strong total and Lewis acid sites compared to other catalysts^30^ and, therefore, is likely to be responsible for the higher GVL selectivity. Therefore, this catalyst allows for obtaining a better synergy between the two oxides, resulting in an optimal BAS/LAS ratio of 0.19 (Table 2), which is beneficial for promoting all stages of the reaction. Indeed, TEM analysis of this material exhibited improved dispersion of Sn and Zr,^30^ potentially leading to the presence of Lewis acid sites and increased Bro̷nsted acidity associated with the zeolite. However, there was always a certain amount of IPL that cannot be converted into GVL, confirming it as the most challenging step of the process.

Overall, comparing the results with the ones obtained in batch mode by Garca et al., the operations under continuous flow conditions resulted in lower selectivities toward GVL and FPE and higher selectivities toward AnL and IPL. Additionally, the formation of undesired byproducts appeared to be more pronounced in continuous mode. These differences may be attributed to variations in the hourly space velocity, which could favor the progression of the overall reaction under batch conditions. Nonetheless, in both operational modes, the bimetallic catalyst demonstrated the best overall performance.

The results of the catalytic tests in continuous flow show that the selectivity of products was influenced by the composition of the catalyst and thus by the acid properties of the chosen catalytic system. To fully understand the results obtained, the selectivity in GVL was plotted against the LAS values for the supported systems (Figure 7).

**Figure 7:**
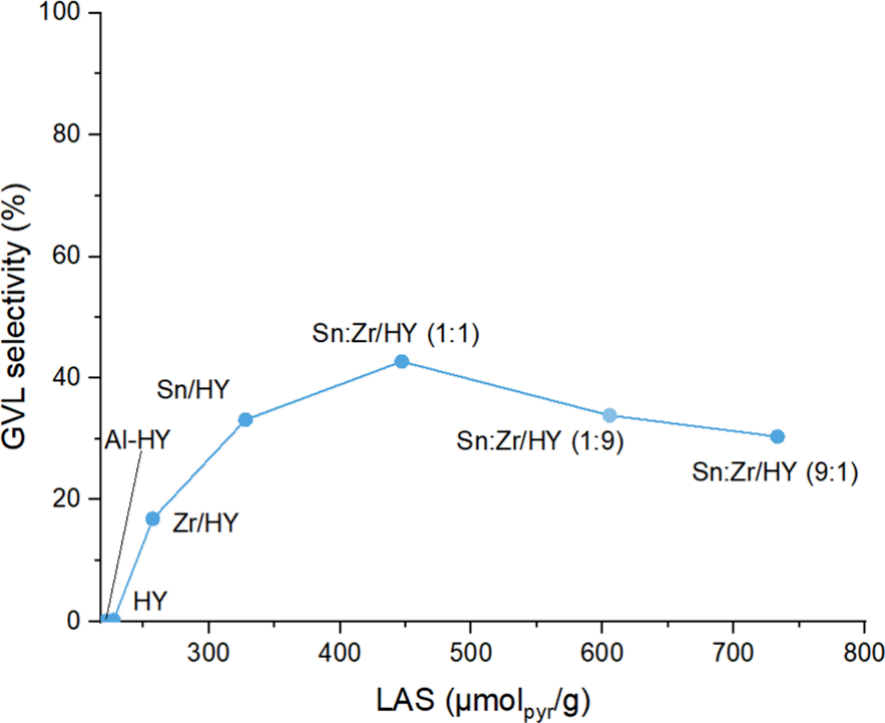
Selectivity trend of GVL vs Lewis’s density acid sites.

The selectivity followed a “volcano plot” trend as a function of LAS. Starting from the zeolites, which had insufficient Lewis acidity, there is no yield in GVL, and proceeding with the supported catalysts, the trend increased. By incorporating only Zr or Sn, the number of Lewis acid sites increased to 257.2 μmol g^–1^ and 327.9 μmol g^–1^ and also the GVL selectivity, from zero, increased to 17% and 35%. A maximum in the trend was obtained with the Sn:Zr/HY catalyst (1:1), which has 447.01 μmol g^–1^ of Lewis acid sites and was the most promising material with a GVL yield of 45%, As the number of Lewis acid sites continued to increase, a decreasing trend was observed, in fact that the catalyst (1:9) showed a GVL selectivity of 35% with 605.5 μmol g^–1^ Lewis acid sites and the catalyst (9:1) had a GVL selectivity of 30% with 733.3 μmol g^–1^ Lewis acid sites. This trend suggests that there is a composition that leads to the optimal number of Lewis acid sites, beyond which the balance between the different reaction steps makes the abundance of those sites unnecessary.

### 3.2 Study of the Reaction Mechanism

To better comprehend the reaction pathway that leads from FU to GVL under the conditions used, the reaction mechanism was evaluated by using the principal intermediates identified in the previous tests as substrates. Four tests were then carried out on Sn:Zr/HY(1:1) starting from FAL, furfuryl ethyl ether (FEE), propyl levulinate (PL), and AnL (Figures S20–S23). Instead of FPE and IPL, not available commercially, FEE and PL were used as they are the most similar molecules to the real intermediates on the market. Every catalytic test lasted about 7 h to obtain stable results and verify the possible deactivation of the catalyst in the presence of a specific intermediate, as we have previously reported with our methodology.^8,33^ Figure 8 shows the average results obtained.

**Figure 8:**
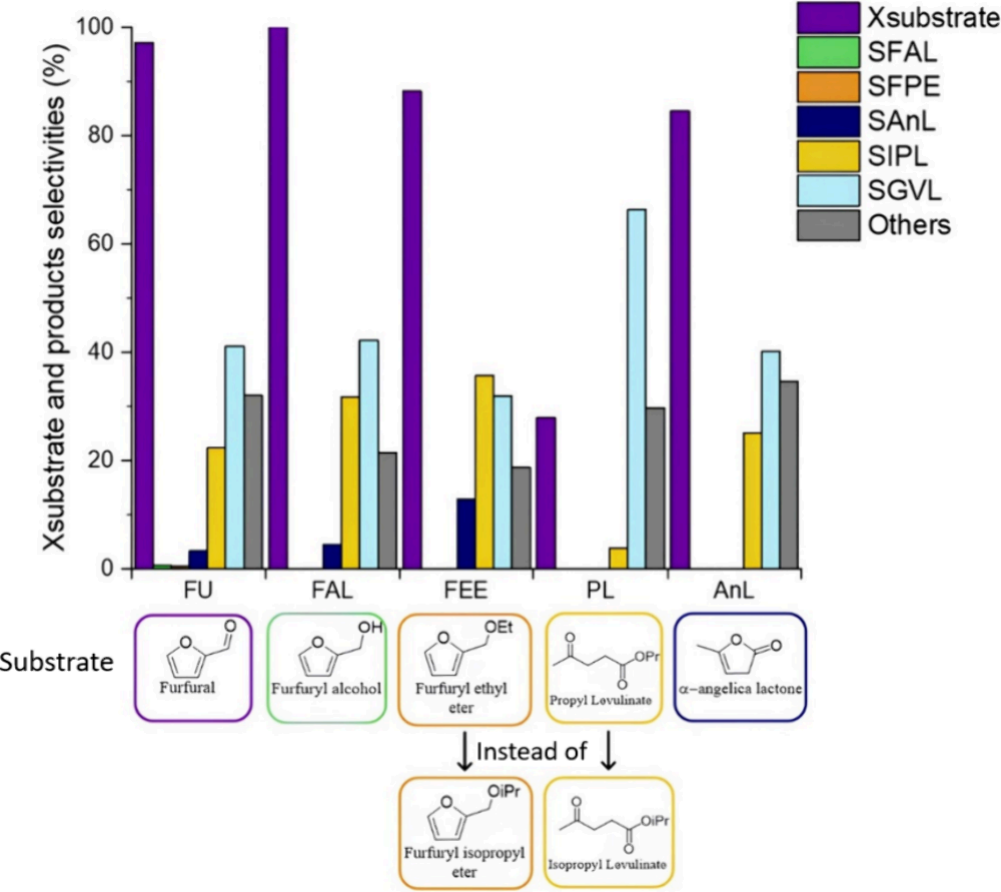
Comparison between substrate conversion and selectivity of products obtained using the principal intermediates as substrates for the production of GVL on Sn:Zr/HY (1:1). Reaction conditions: [substrate] = 67 mM; τ = 10 min, *T* = 180 °C, mcat = 0.47g.

Results obtained using FU and FAL as reagents are similar, consistent with the expectations, given the complete conversion of FU and the absence of selectivity in FAL in the first test. The main difference between these two experiments was the higher production of “Others” obtained starting from the aldehyde, due to its intrinsically higher instability, which favored side reactions. This result confirms the ease of conversion of FU and the presence of a rate-determining step further down the cascade process.

FEE conversion was lower than that of the previous substrates, even if relatively high. It has been previously demonstrated how difficult the conversion of this molecule to the levulinate could be because of the strong Bro̷nsted acidity required.^8,33^ However, probably because of the important contribution of the zeolite’s Bro̷nsted acid sites, in this case, FEE conversion reached 88%. Furthermore, the highest selectivities in AnL and in IPL were detectable, respectively, ca. 15% and 30%, together with the lowest “Others” production under 20%. The presence of a relatively high AnL selectivity suggests the possibility of an interconversion pathway between the two species, as previously reported.^43^ This is further reinforced by the absence of ethyl levulinate, which would be the product of hydrolytic ring opening of FEE. While its absence could be explained by a subsequent fast conversion of the levulinate, this hypothesis can be discarded based on the results obtained using PL as the substrate. Indeed, the transesterification reaction of PL into IPL did not seem to be favored, as only 4% selectivity in IPL was detected at a low conversion of 28%. Hence, ethyl levulinate transesterification into IPL would probably be disfavored too. These results suggest that FEE more easily hydrolyzed to the lactone, which subsequently formed IPL and GVL (Figure 3). In conclusion, despite the high conversion of FEE, its opening to form IPL, avoiding the formation of AnL, proves to be challenging also in the presence of strong Bro̷nsted acidic sites.

AnL conversion was high as well, reaching 85%. It mostly formed GVL, but a relatively high selectivity in IPL was also present. The latter could be justified by the fact that AnL and IPL are bonded by an equilibrium that mostly favors the formation of the levulinate, given the high concentration of iPrOH, used not only as H-donor, but also as solvent. Furthermore, the test performed working with PL demonstrated, as expected, observing at all the performed experiments, the difficulty of its conversion (*X*_PL_ = 28%) in these reaction conditions. This is probably due to the lack of strong Lewis acidic sites, as the FTIR-py analyses on Sn:Zr/HY (1:1) demonstrated.^30^

### Long-Term Stability

After each reaction, the spent catalysts were characterized using XRD analysis and N_2_ adsorption–desorption analysis to assess whether the reaction led to changes in the physicochemical properties of the studied catalysts. As an example, XRD analysis of catalyst Sn:Zr/HY (1:1) is provided in Figure 9a. From the comparison of XRD analyses of the Sn:Zr/HY (1:1) catalyst before (fresh) and after the reaction (spent), no significant differences are noticeable; even in the spent catalyst, the characteristic diffraction peaks of the oxides remain indistinguishable, and the typical diffraction peaks of zeolite Y were still visible, albeit with lower intensity, indicating a reduced crystallinity of the zeolitic structure. Therefore, it can be concluded that the reaction did not induce significant structural modifications to the zeolite. For all catalysts, including those where the pattern of their respective supported oxides was visible, no significant structural modifications were observed after the reaction.

**Figure 9:**
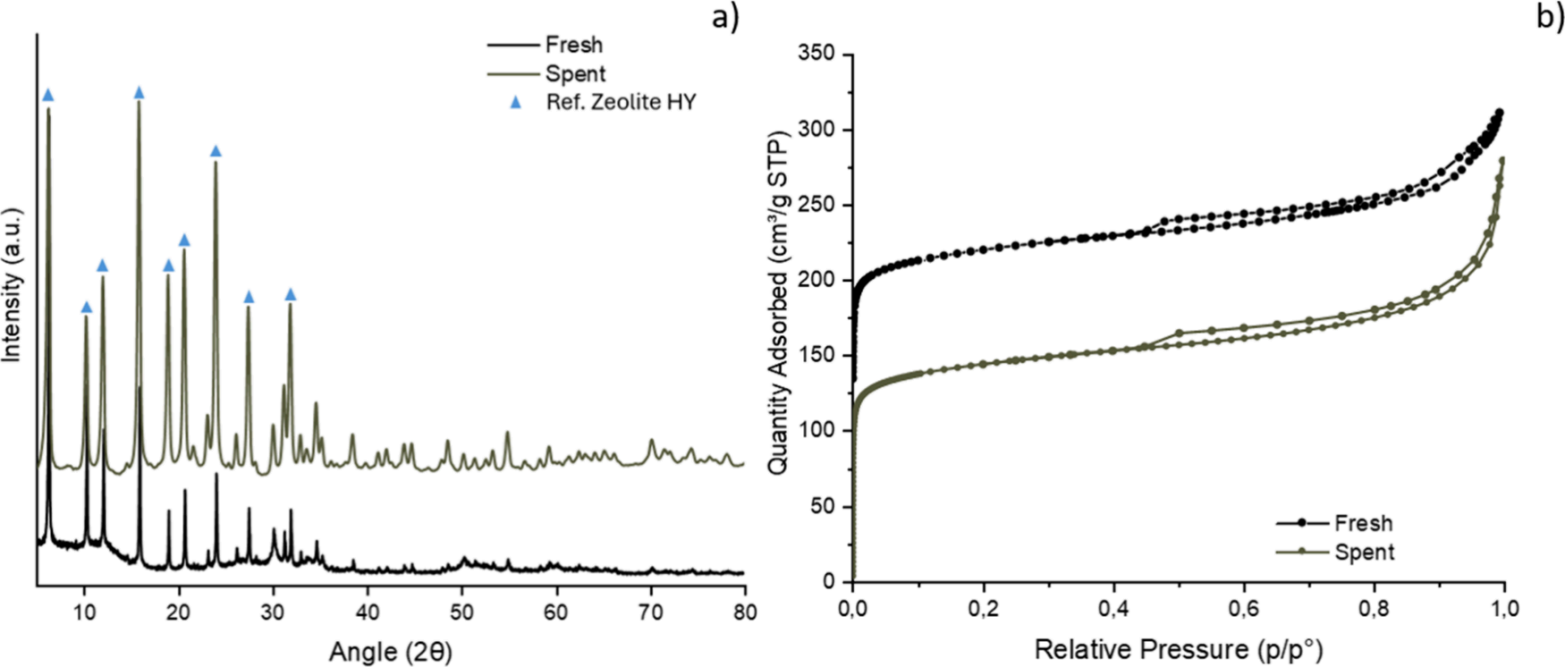
(a) XRD patterns of fresh and spent Sn:Zr/HY (1:1); (b) N_2_ adsorption–desorption isotherms of fresh and spent Sn:Zr/HY (1:1).

In Figure 9b, a comparison between the isotherms obtained from the N_2_ adsorption–desorption analyses of Sn:Zr/HY (1:1) before and after the reaction can be observed. The curve remains of type IV with H4 hysteresis, and the amount of adsorbed N_2_ postreaction was significantly lower, as well as the surface area, which decreased from 730 to 592 m^2^/g. This can be attributed to the deposition of organic species that formed during the reaction that could block the pores, resulting in a surface area decrease ranging from 20% to 30% for the composite catalysts. This suggests that longer reaction times could potentially lead to fouling of the catalyst as has been previously observed; nevertheless, the tested reaction conditions showed good relative stability of the materials, possibly due to their high specific surface area.

Table 2 reports the summary and comparison of the characterization of spent and fresh catalysts.

As previously discussed, the presence of organic deposits on the surface of the catalysts (as inferred from the decrease in specific surface area) suggests that the increase in time on stream could potentially lead to fouling. To assess the limits of catalyst stability, longer-duration tests were conducted on the Sn:Zr/HY (9:1) and regenerated Sn:Zr/HY (9:1) systems.

Finally, in Figure 10, the long-term stability test of the catalyst Sn:Zr/HY (9:1) is reported, which is essential for the development of effective catalysts. As previously reported, a 30% maximum in GVL selectivity was achieved in the initial hours of the reaction. IPL exhibited a selectivity of 15%, and very small quantities of FPE and FAL were observed too. These values remained stable for about 20 h of reaction; thereafter, the GVL selectivity decreased, stabilizing for another 20 h of reaction at a value of 10%. As the GVL selectivity decreased, the selectivities for IPL, FPE, and AnL increased. This suggests an initial fouling of Lewis acid sites; indeed, the final steps of the cascade reaction to GVL are catalyzed by Lewis acid sites. If these sites are insufficient or deactivated, the reaction cannot proceed efficiently, resulting in the accumulation of intermediates and increased selectivity toward FPE, AnL and IPL. After about 45 h of reaction, the predominant product was FAL, while GVL selectivity decreased to zero. This is indicative of a subsequent fouling of the Bro̷nsted acid sites, which leads to almost complete deactivation of the material. It is crucial to emphasize that this is one of the few long-term studies on catalysts tested in a continuous liquid-flow plant for the conversion of FU to GVL.^8,33^ The only other study performed by a different research group, attempting the same process, observed signs of catalyst deactivation after just 1 h of operation.^40^

**Figure 10:**
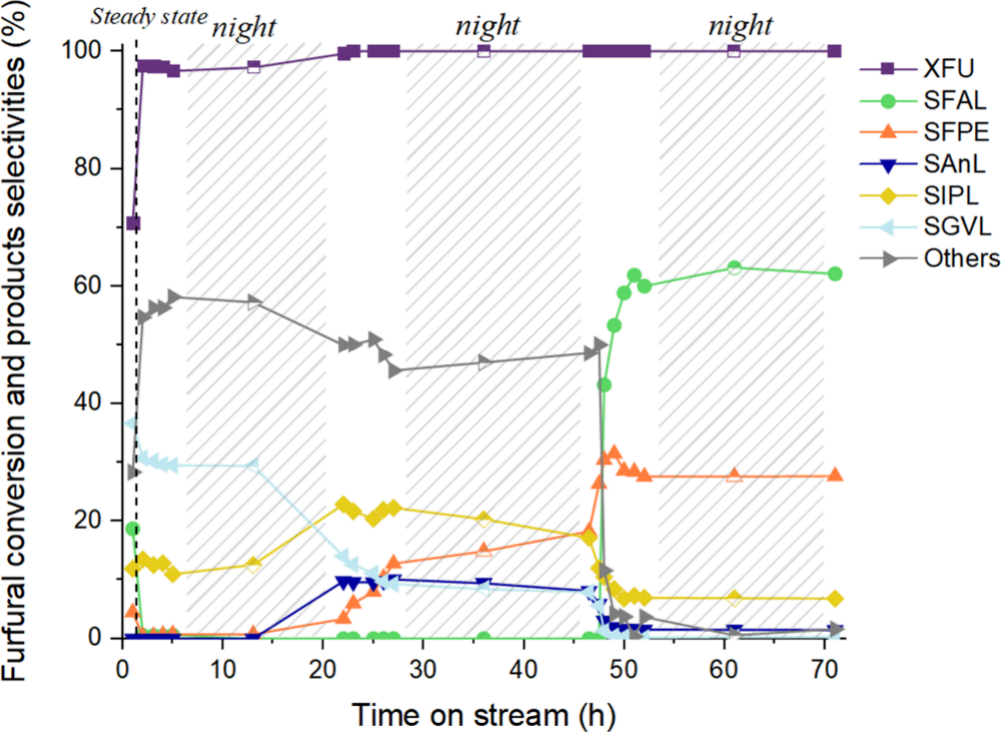
FU conversion and selectivity of products as a function of time (h) on Sn:Zr/HY (9:1). Reaction conditions: [FU] = 67 mM, 1 equiv of H_2_O, τ = 10 min, *T* = 180 °C, mcat = 0.5209 g.

### Regeneration

Catalytic tests revealed a clear deactivation of the tested materials (Figure 10). Probably, carbonaceous residues and/or organic species are formed on the surface of the catalyst, blocking the active sites (Figure 11). This conjecture is partially supported by characterizations, particularly by comparing results obtained from N_2_ adsorption–desorption measurements conducted on the catalysts before and after the tests (Figure 9b). These indicate a decrease in specific surface areas concurrent with an increase in pore volume (Table S1). Given this assumption, it is reasonable to consider that the catalytic activity of the systems could be restored through a thermal regeneration process. The choice of this regeneration method lies in its ability to remove organic species and other carbonaceous residues from the catalyst surface through a moderate to high temperature process.^46^ Therefore, the Sn:Zr/HY (9:1) catalyst was regenerated using a simple and practical thermal treatment (500 °C in static air for 3 h).

**Figure 11:**
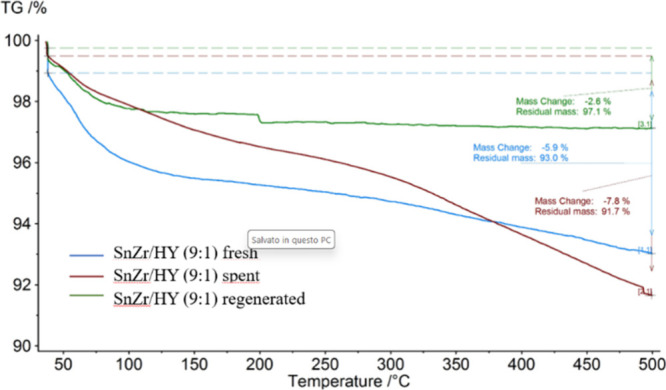
TGA analysis on SnZr/HY catalyst fresh, spent, and after the regeneration.

To verify the success of regeneration, a test under the same reaction conditions previously employed was conducted. The comparison between the average performances of the fresh and regenerated catalysts is reported in Figure 12. As can be observed from the results, the regeneration process was successful. The trend of selectivities suggests that both Lewis and Bro̷nsted acid sites are nearly fully restored, achieving selectivity values in GVL and IPL of 30% and 15%, respectively, comparable to the values obtained when working with the fresh catalyst in the initial test. The thermal treatment removes the organic species on the catalyst’s surface (Figure 11), restoring accessibility to the acid sites, while the chemical properties are not affected.

**Figure 12:**
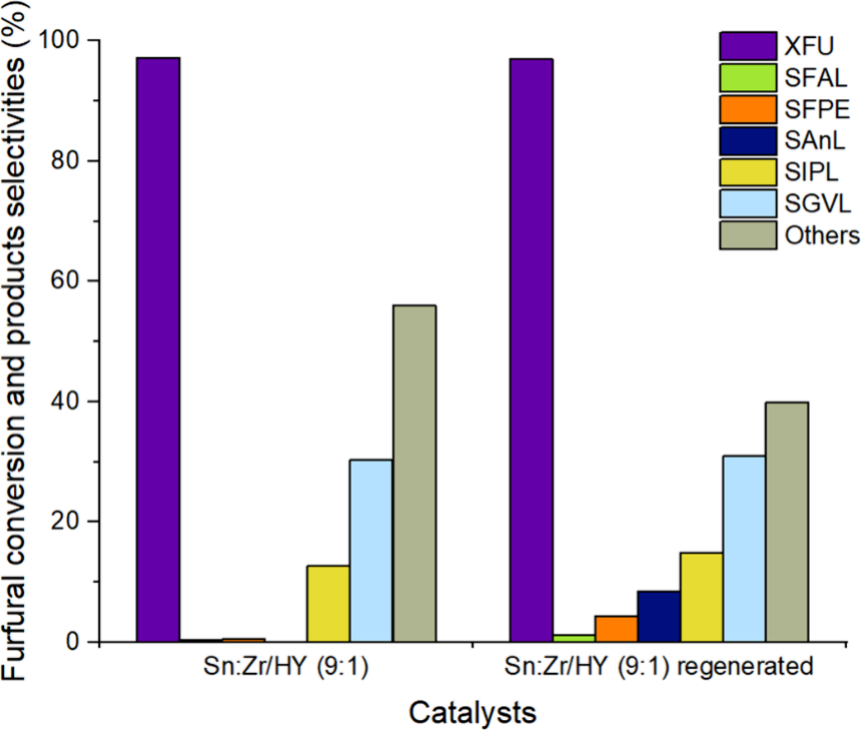
Comparison of the performance of Sn:Zr/HY (9:1) and regenerated Sn:Zr/HY (9:1) catalysts in the FU to GVL cascade reaction; the conversion of FU is >90% in each case. Reaction conditions: [FU] = 67 mM, 1 equiv of H_2_O, τ = 10 min, *T* = 180 °C, time on stream = 6h.

An additional stability test was conducted on the regenerated Sn:Zr/HY (9:1) catalyst, which lasted approximately 50 h to assess the catalyst’s long-term stability after thermal treatment (Figure 13).

**Figure 13:**
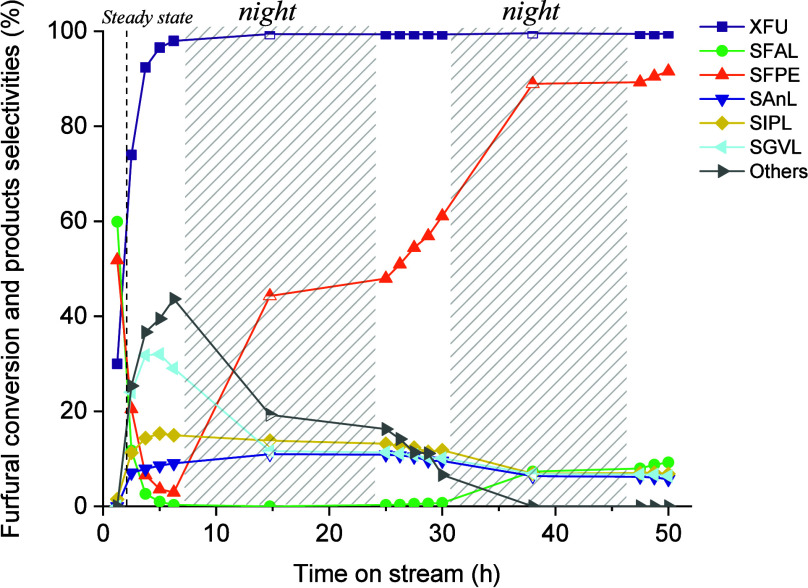
Trend of FU conversion and selectivity of products as a function of time (h) on regenerated Sn:Zr/HY (9:1). Reaction conditions: [FU] = 67 mM, 1 equiv of H_2_O, τ=10 min, *T* = 180 °C, mcat = 0.4500 g.

As previously seen in the regeneration section, during the initial hours of the reaction following the regeneration process, the catalyst attained the same catalytic activity as the fresh catalyst, reaching 30% selectivity in GVL. However, the deactivation of the material after the regeneration process occurred more rapidly, indicating lower stability as a function of time. While a similar deactivation of the Lewis acid sites can be observed initially, the GVL selectivity does not reach zero, and the deactivation of the Bro̷nsted acid sites was only partial, leading to an FPE selectivity of ∼92% and contemporaneously avoiding the formation of “Others”.

In the end, the comparison of the best results obtained in this work with the ones reported in the literature is shown. The STY (mmol_GVL_ * g^–1^ * h^–1^) obtained with Sn:Zr/HY (1:1) is the highest ever obtained in the case of FU conversion to GVL in liquid-phase continuous flow mode, probably due to the high dispersion of the two metals on the zeolite support, which allows a high number of available active sites, and because of an optimal BAS/LAS ratio obtained thanks to the zeolite dealumination. The reaction conditions for each study are listed in Table 3.

**Table 3:** Comparison between the GVL Space Time Yield (mmol_GVL_* g_CAT_^–1^ * h^–1^) Achieved in This Work in Comparison to the Results Published in the Scientific Literature on Other Continuous Flow Systems and Batch for the Same Reaction

catalyst	[furfural] (mM)	reaction temperature (°C)	GVL space time yield (mmol_GVL_* g_CAT_^–1^ * h^–1^)	reference
Sn:Zr/HY (1:1) cont	67	180	1.470	this work
Sn:Zr/HY (1:1) batch	50	180	0,975	^30^
1Pt/Sep + 10Zr/Sep	67	180	0.216	^33^
Ti/Zr/O (1:1)	67	180	0.372	^8^
Zr–Al–beta	62	150	0.090	^40^

Moreover, continuous flow STY (mmol_GVL_ * g^–1^ * h^–1^) results favorable even when compared to the batch one, demonstrating the higher efficiency of the process chosen (Figure 14).

**Figure 14:**
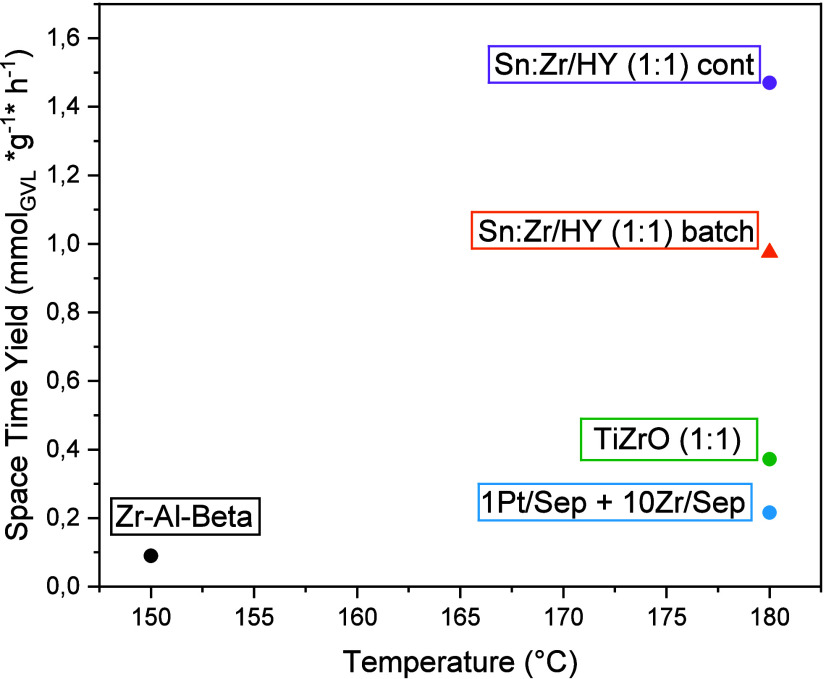
Comparison between the GVL space time yield (mmol_GVL_* g_CAT_^–1^ * h^–1^) achieved in this work in comparison to the results published in the scientific literature on other continuous flow systems (circle solid) and batch systems (triangle up solid). The labels refer to the entries in Table 3.

## Conclusions

The catalytic systems, tested in a liquid-phase continuous flow reactor, were based on Sn and Zr oxides, with varying metal ratios, supported on a dealuminated Y-zeolite (HY). The Sn:Zr/HY (1:1) system was identified as the optimal catalyst for the cascade reaction from FU to GVL via the CTH mechanism. This catalyst exhibited the best balance of Bro̷nsted and Lewis acid sites (BAS/LAS), enabling efficient catalysis of all reaction steps and achieving a yield of approximately 15% in IPL and 45% in the desired product as GVL. It has also been demonstrated that Sn:Zr-based catalysts could be easily recovered and reused, achieving conversions and selectivity close to the initial ones. Furthermore, a mechanism for the cascade transformation from FU to GVL was proposed based on the roles of Lewis and Bro̷nsted acid catalysts. The study examining the correlation between Lewis acidity and selectivity in GVL revealed a maximum in the trend, corresponding to the most promising system, Sn:Zr/HY (1:1). Finally, the possibility of reversing the fouling of the catalysts via thermal regeneration was demonstrated. Finally, the STY (mmol_GVL_ * g^–1^ * h^–1^) obtained with the latter resulted to be the highest reported in the literature for liquid-phase continuous flow processes and higher than the one obtained working with the same material in batch mode.

## Supplementary Material



## Notes

N_2_ adsorption desorption isotherms, pore distributions, and XRD diffractograms for all the fresh and spent catalysts; FU conversion and selectivity of products as a function of time (h) for all the catalysts (PDF)
