# Supplementary material for: Furfural Valorization to γ‑Valerolactone over Zr/Sn Zeolite-Supported Catalysts in a Liquid-Phase Continuous Flow Reactor
Source: Energy Fuels. 2025 Dec 23;40(1):478–89. doi: 10.1021/acs.energyfuels.5c05628 (PMC12797859; doi:10.1021/acs.energyfuels.5c05628)
Supplement: Supplementary file 1 [file ef5c05628_si_001.pdf]

SUPPORTING INFORMATION

Furfural valorization to γ-valerolactone on Zr/Sn zeolite-supported catalysts in a liquid-phase continuous-flow reactor

Vittoria Saraceni[1], Anna Saotta[1], Adrián García[2], Alessandro Allegri[1], Giuseppe Fornasari[1], Benjamin. Solsona[2], Nikolaos Dimitratos[1*], Stefania Albonetti[1*]*

[1]Department of Industrial Chemistry "Toso Montanari", Center for Chemical Catalysis – C3, Alma Mater Studiorum – Università di Bologna, Via Piero Gobetti 85, Bologna 40129, Italy

[2]Department of Chemical Engineering (ETSE), Universitat de València, Av. Universitat s/n, Burjassot-Valencia 46100, Spain

* alessandro.allegri2@unibo.it; nikolaos.dimitratos@unibo.it; stefania.albonetti@unibo.it

Fresh characterization

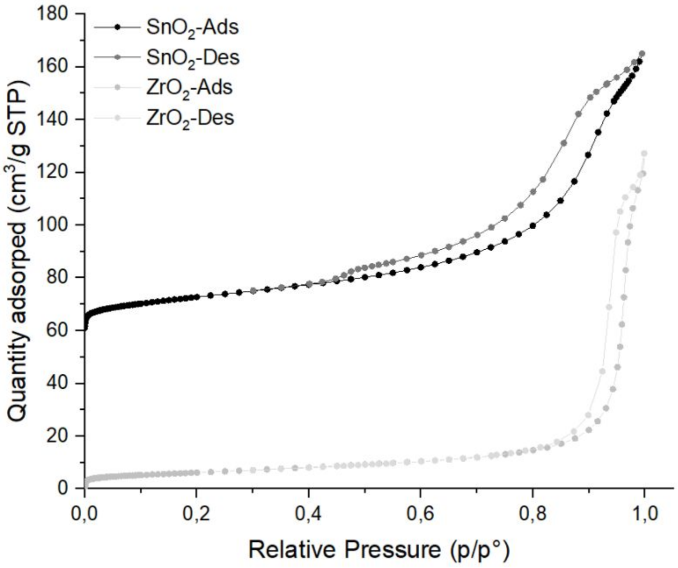

Figure S1. N_2 adsorption and desorption isotherms of SnO_2 and ZrO_2.

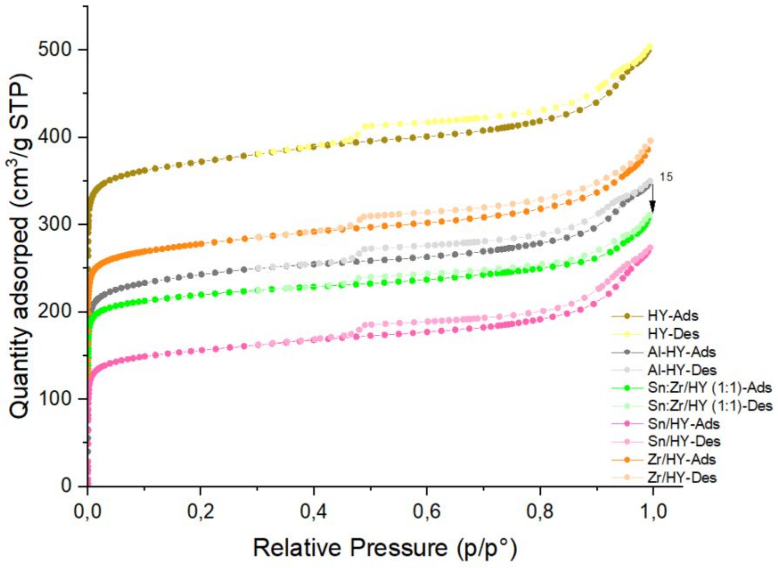

Figure S2. N_2 adsorption and desorption isotherms for Sn and Zr-based catalysts supported on dealuminated zeolite, HY, and for Al-HY and HY zeolites.

Table S1. Porosity characterization for the fresh and spent catalysts.

Catalyst	Pore volume *fresh* *(cm³/g)*	Pore volume spent *(cm³/g)*	Pore diameter fresh *(Å)*	Pore diameter spent *(Å)*
HY	0.48	0.54	60	123
Sn/HY	0.37	0.38	69	125
Sn:Zr/HY (9:1)	0.35	-	72	-
Sn:Zr/HY (1:1)	0.34	0.41	61	138
Sn:Zr/HY (1:9)	0.39	-	61	-

Zr/HY	0.39	0.46	62	128
SnO_2	0.06	0.15	229	204
ZrO_2	0.14	0.16	85	85

Catalytic tests

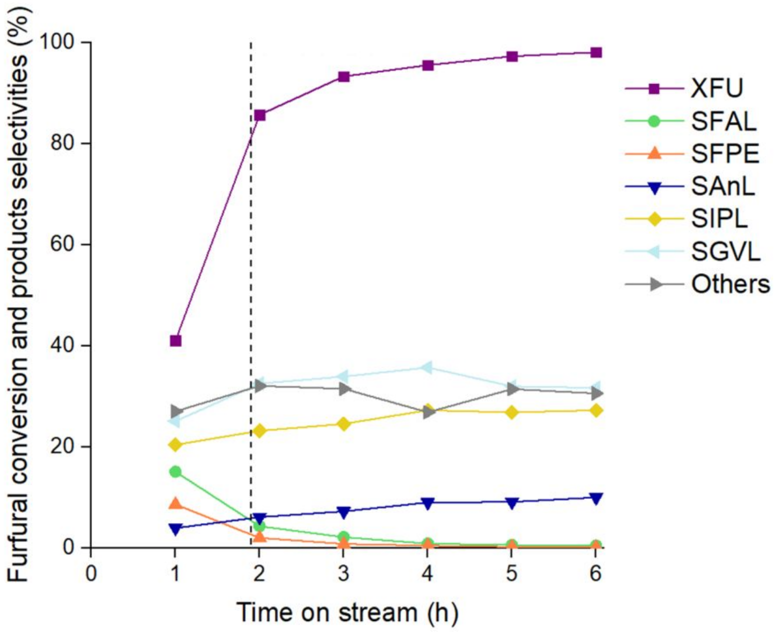

Figure S3. FU conversion and selectivity of products as a function of time (h) on Sn/HY. Reaction conditions: [FU]=67 mM, 1 eq H_2O, τ=10 min, T=180 ˚C, mcat= 0.5194 g.

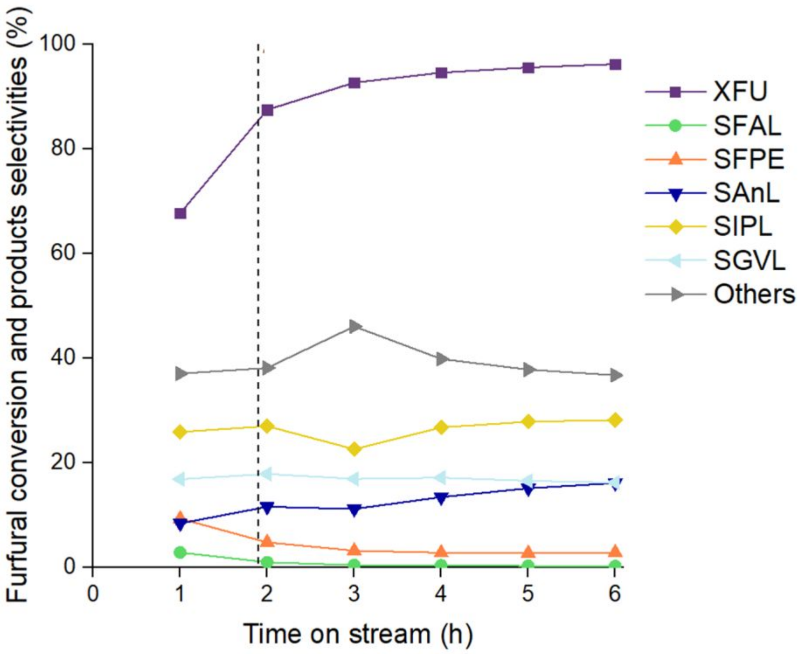

Figure S4. FU conversion and selectivity of products as a function of time (h) on Zr/HY. Reaction conditions: [FU]=67 mM, 1 eq H_2O, τ=10 min, T=180 ˚C, mcat= 0.4792 g.

Comparison between fresh and spent characterization

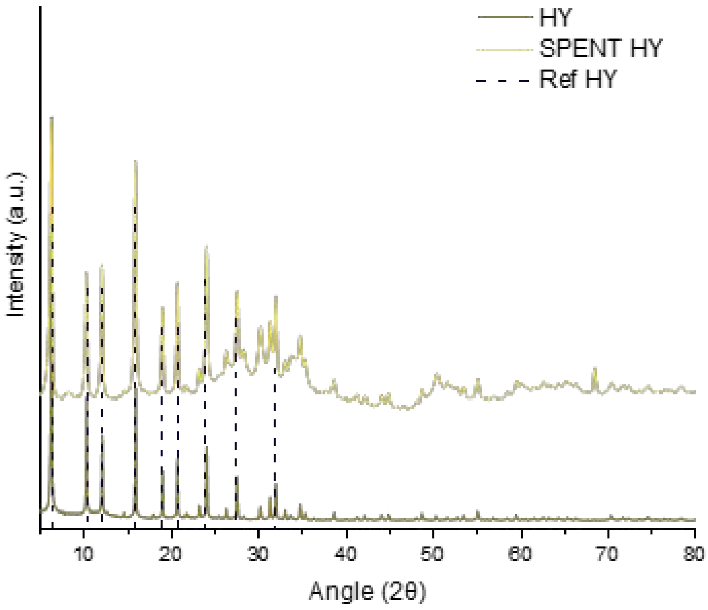

Figure S5. XRD patterns for HY fresh and spent catalysts.

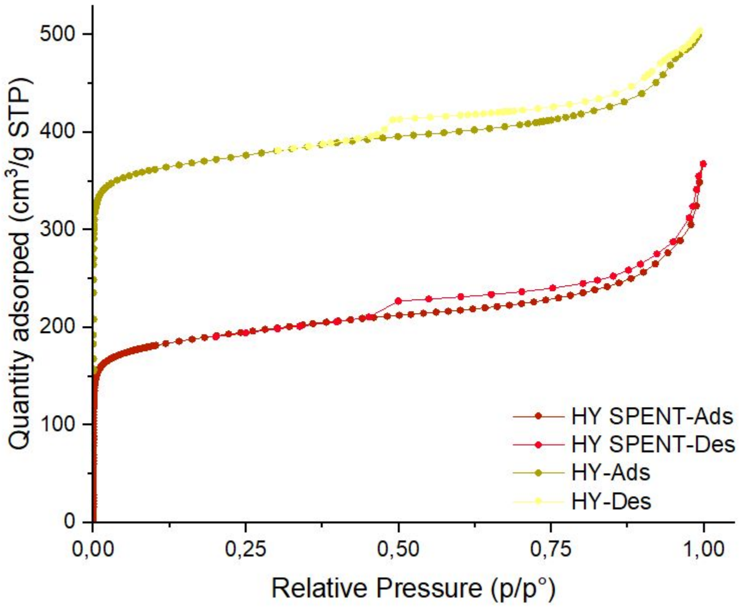

Figure S6. N_2 adsorption and desorption isotherms for HY fresh and spent catalysts.

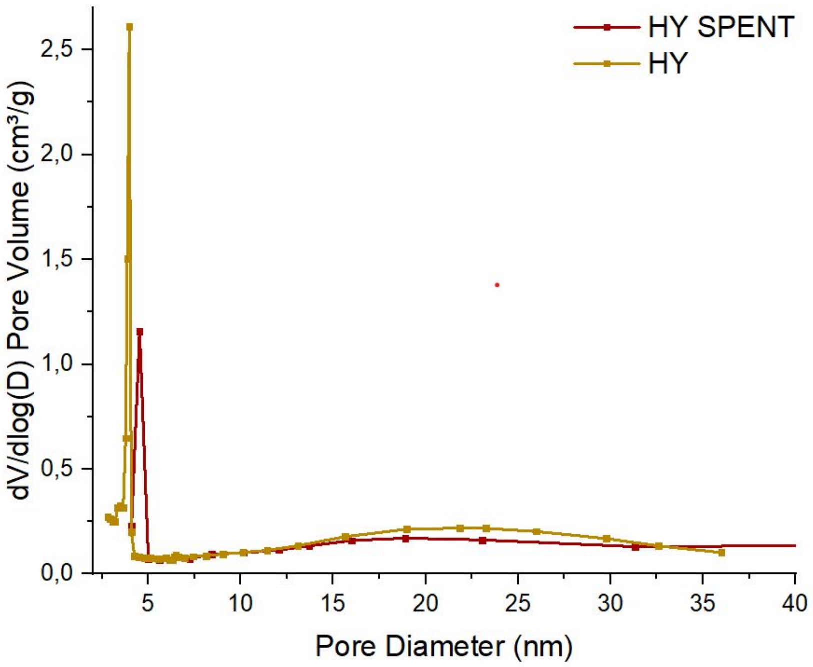

Figure S7. Pore size distribution for HY fresh and spent catalysts.

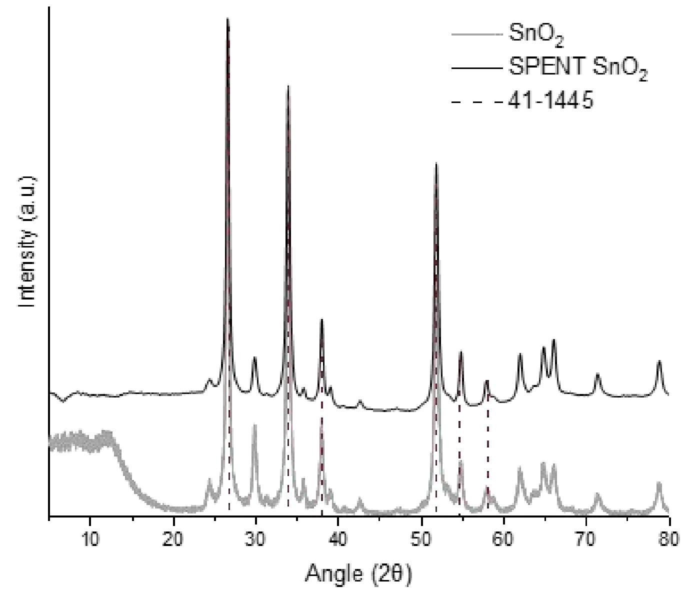

Figure S8. XRD patterns for SnO_2 fresh and spent catalysts.

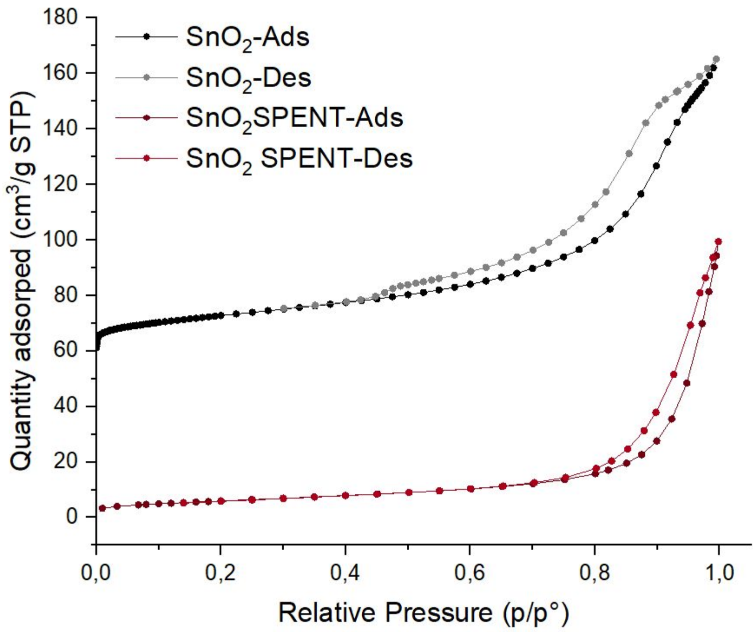

Figure S9. N_2 adsorption and desorption isotherms for SnO_2 fresh and spent catalysts.

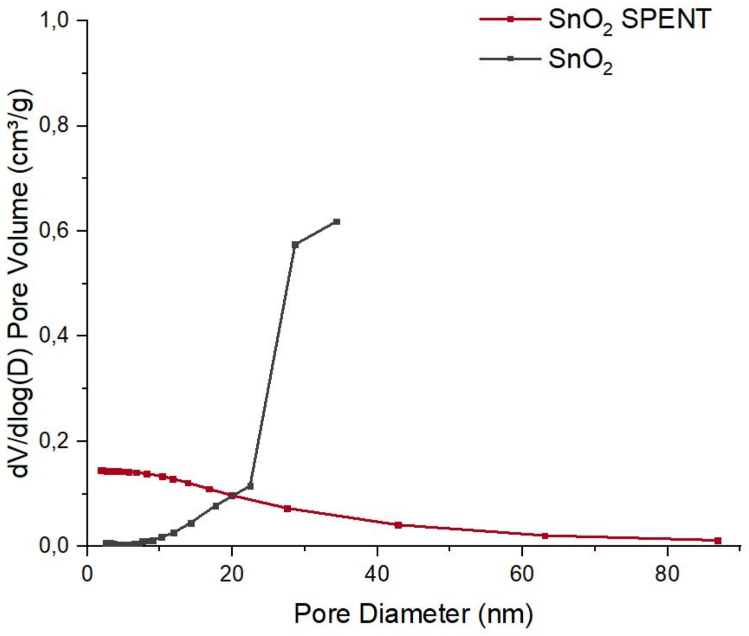

Figure S10. Pore size distribution for SnO_2 fresh and spent catalysts.

ZrO_2

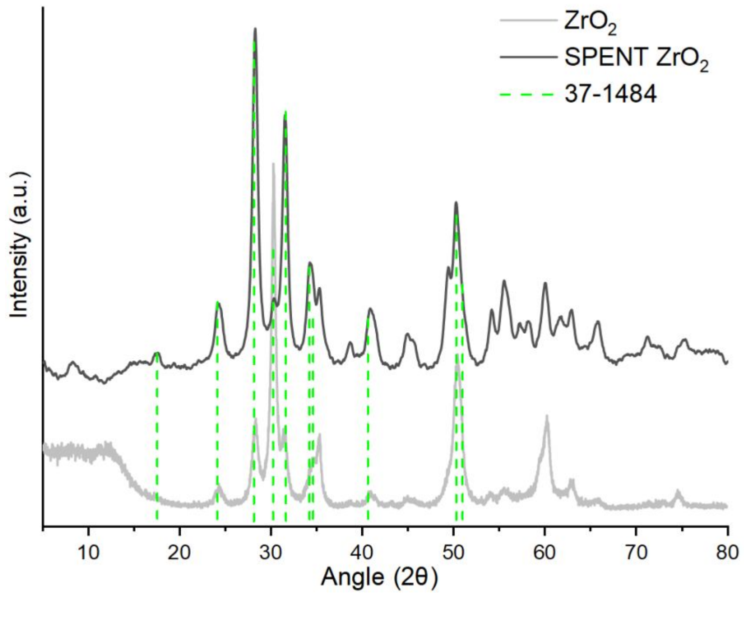

Figure S11. XRD patterns for ZrO_2 fresh and spent catalysts.

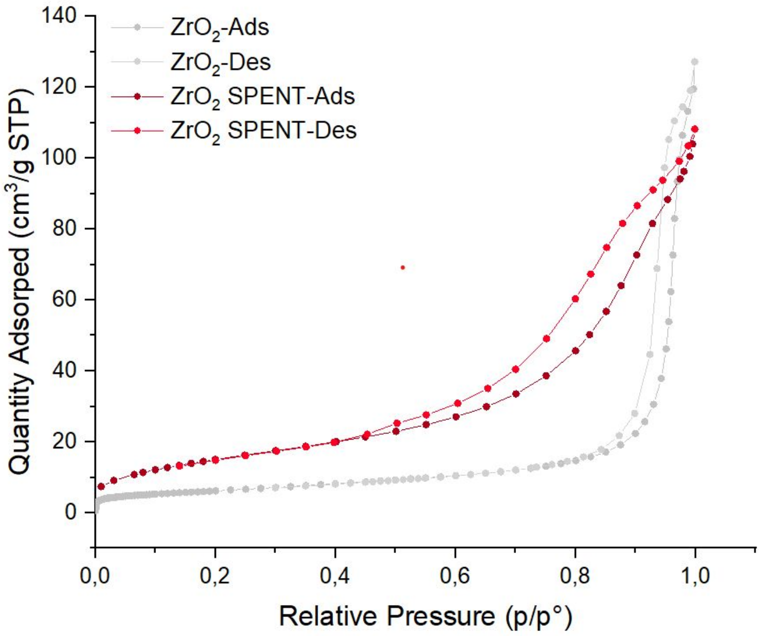

Figure S12. N_2 adsorption and desorption isotherms for ZrO_2 fresh and spent catalysts.

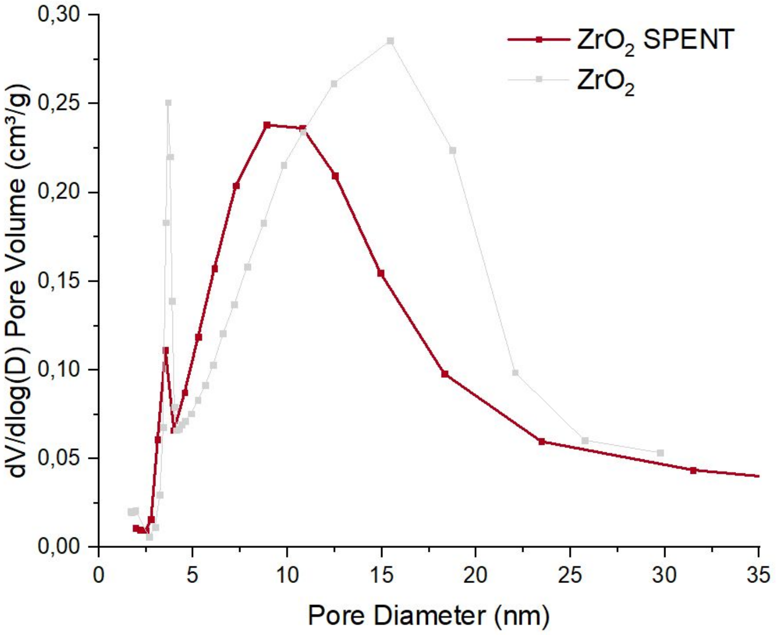

Figure S13. Pore size distribution for SnO_2 fresh and spent catalysts.

Sn/HY

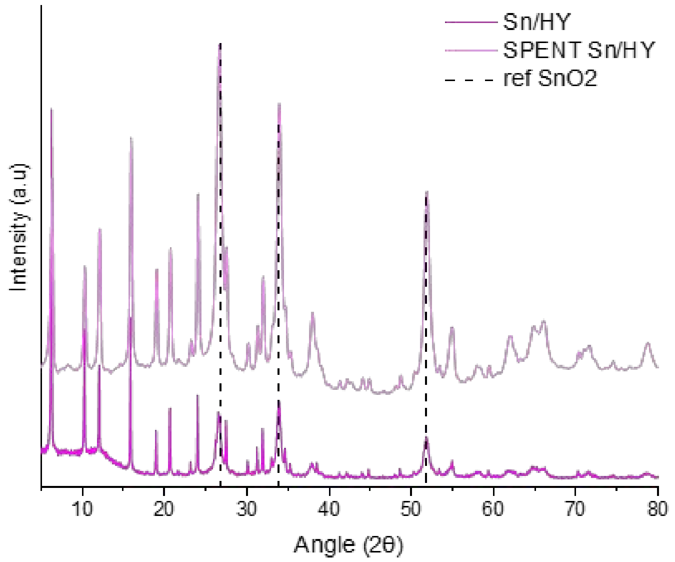

Figure S14. XRD patterns for Sn/HY fresh and spent catalysts.

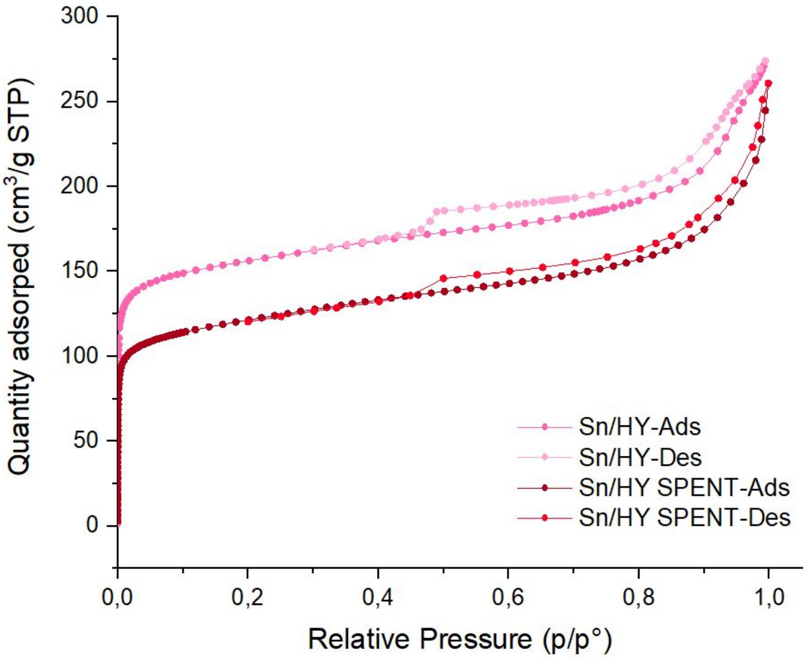

Figure S15. N_2 adsorption and desorption isotherms for Sn/HY fresh and spent catalysts.

Zr/HY

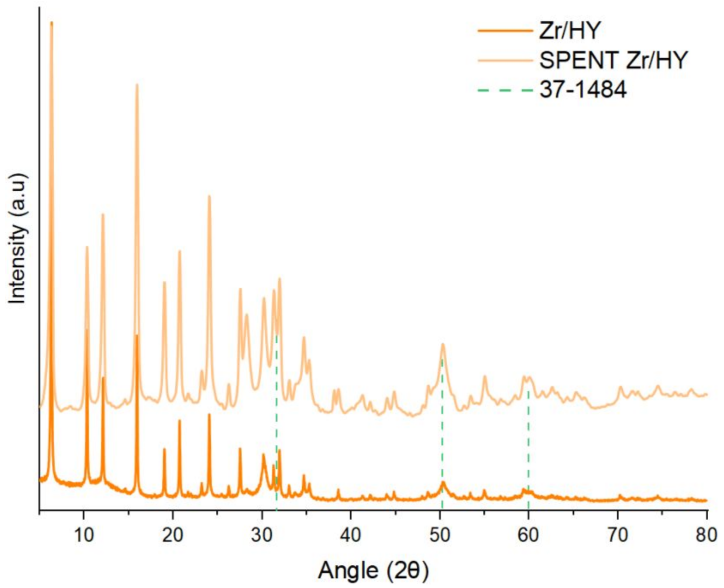

Figure S16. XRD patterns for Zr/HY fresh and spent catalysts.

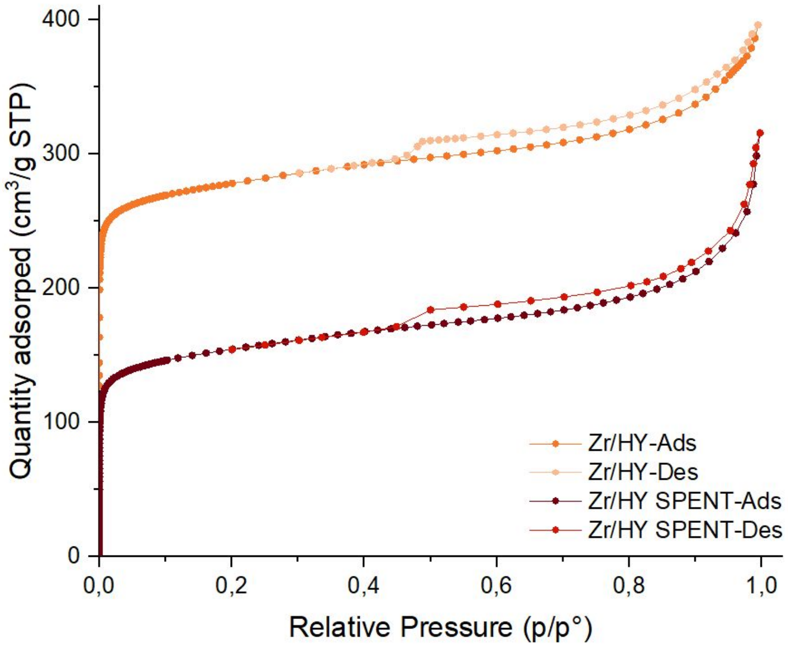

Figure S17. N_2 adsorption and desorption isotherms for Zr/HY fresh and spent catalysts.

Sn:Zr/HY (9:1)

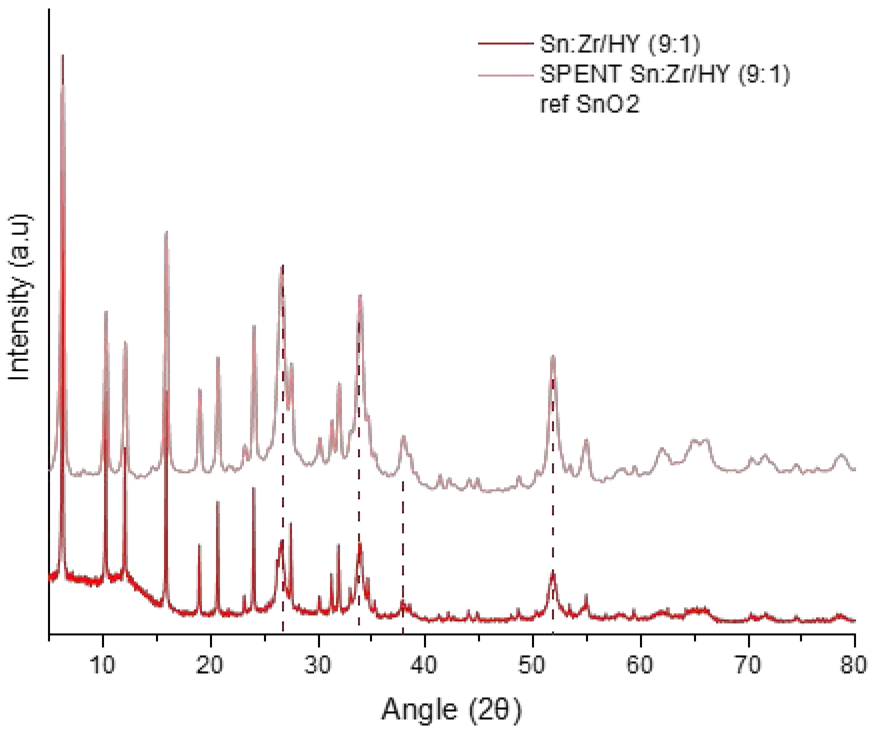

Figure S18. XRD patterns for Sn:Zr/HY (9:1) fresh and spent catalysts.

Sn:Zr/HY (1:9)

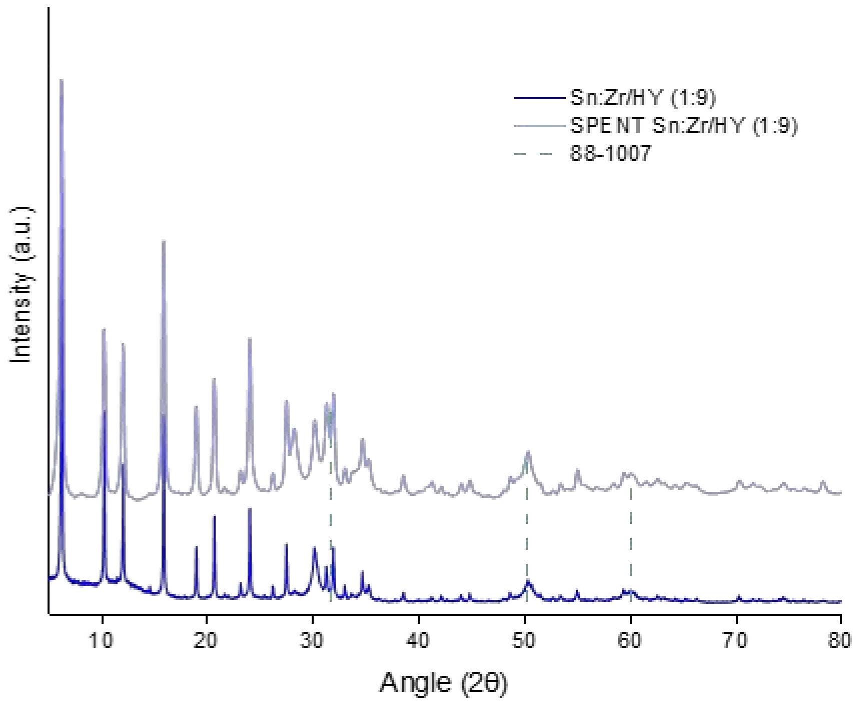

Figure S19. XRD patterns for Sn:Zr/HY (1:9) fresh and spent catalysts.

Study of the reaction mechanism

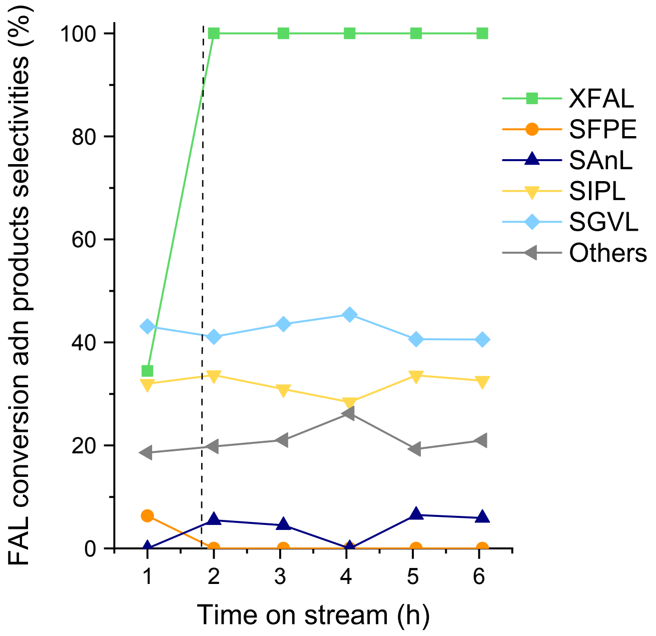

Figure S20. FAL conversion and selectivity of products as a function of time (h) on Sn:Zr/HY (1:1). Reaction conditions: [FAL]=67 mM, 1 eq H_2O, τ=10 min, T=180 ˚C, mcat = 0.47 g.

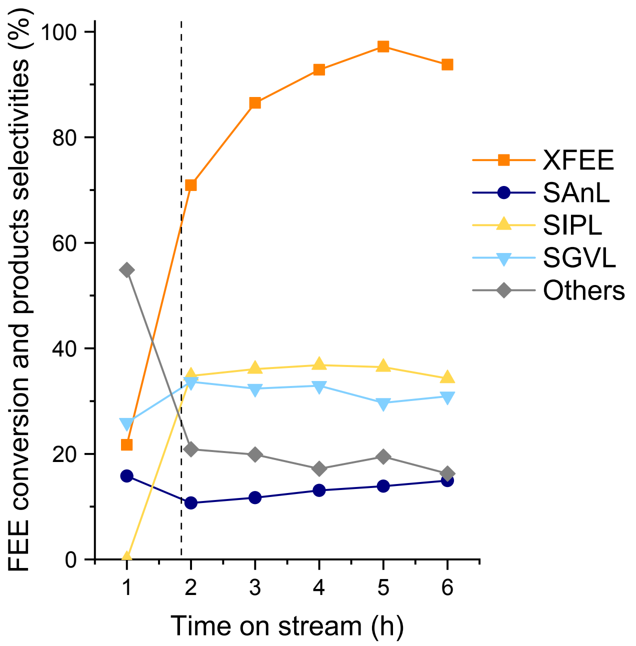

Figure S21. FEE conversion and selectivity of products as a function of time (h) on Sn:Zr/HY (1:1). Reaction conditions: [FEE]=67 mM, 1 eq H_2O, τ=10 min, T=180 ˚C, mcat = 0.47.

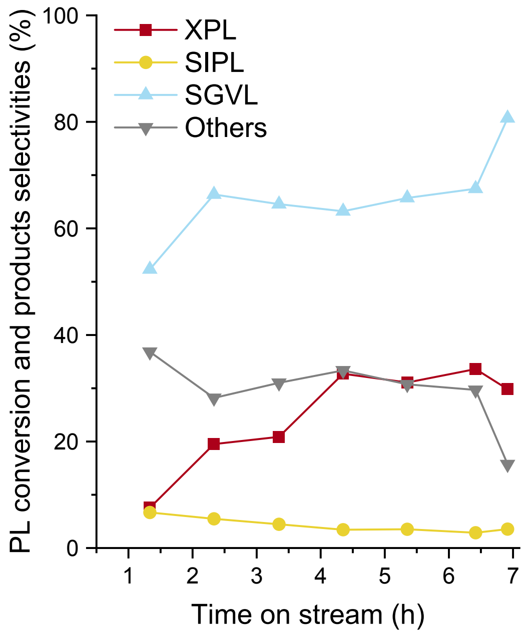

Figure S22. IPL conversion and selectivity of products as a function of time (h) on Sn:Zr/HY (1:1). Reaction conditions: [IPL]=67 mM, 1 eq H_2O, τ=10 min, T=180 ˚C, mcat = 0.47 g.

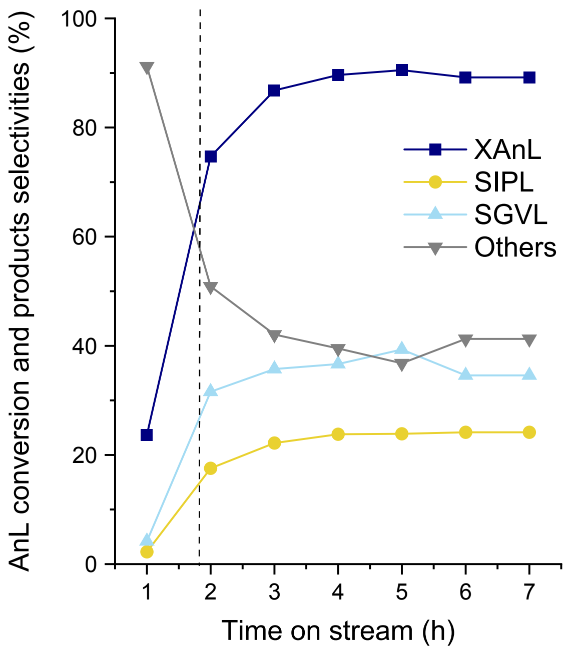

Figure S23. Trends of AnL conversion and selectivity of products as a function of time (h) on Sn:Zr/HY (1:1). Reaction conditions: [AnL]=67 mM, 1 eq H_2O, τ=10 min, T=180 ˚C, mcat = 0.47.

ASSOCIATED CONTENT

Data availability

The data underlying this study are openly available in "*Data on the valorization of furfural to γ-valerolactone in liquid-phase continuous-flow over Zr/Sn zeolite-supported catalysts*" at https://doi.org/10.6092/unibo/amsacta/8555
